# HIV, STI and renal function testing frequency and STI history among current users of self-funded HIV pre-exposure prophylaxis, a cross-sectional study, Germany, 2018 and 2019

**DOI:** 10.2807/1560-7917.ES.2022.27.14.2100503

**Published:** 2022-04-07

**Authors:** Uwe Koppe, Janna Seifried, Ulrich Marcus, Stefan Albrecht, Klaus Jansen, Heiko Jessen, Barbara Gunsenheimer-Bartmeyer, Viviane Bremer

**Affiliations:** 1 Department of Infectious Disease Epidemiology, Robert Koch Institute, Berlin, Germany; 2 Department of Epidemiology and Health Monitoring, Robert Koch Institute, Berlin, Germany; 3 Praxis Jessen^2^ und Kollegen, Berlin, Germany

**Keywords:** HIV Pre-exposure prophylaxis, men who have sex with men, HIV testing, STI testing, STI history, kidney function

## Abstract

**Introduction:**

Users of pre-exposure prophylaxis (PrEP) require periodic testing for HIV, sexually transmitted infections (STI) and renal function. Before PrEP was made free of charge through statutory health insurance in late 2019, PrEP users in Germany had to pay for testing themselves.

**Aim:**

We investigated self-reported HIV, STI and renal function testing frequencies among self-funded PrEP users in Germany, factors associated with infrequent testing, and STI diagnoses.

**Methods:**

A cross-sectional anonymous online survey in 2018 and 2019 recruited current PrEP users via dating apps for men who have sex with men (MSM), a PrEP community website, anonymous testing sites and friends. We used descriptive methods and logistic regression for analysis.

**Results:**

We recruited 4,848 current PrEP users. Median age was 37 years (interquartile range (IQR): 30–45), 88.7% identified as male, and respectively 26.3%, 20.9% and 29.2% were tested less frequently for HIV, STI and renal function than recommended. Participants with lower STI testing frequency were significantly less likely to report STI diagnoses during PrEP use, especially among those with many partners and inconsistent condom use. Factors most strongly associated with infrequent testing included not getting tested before starting PrEP, using PrEP from informal sources and on-demand/intermittent PrEP use.

**Discussion:**

In a setting of self-funded PrEP, many users obtained medical tests less frequently than recommended, which can lead to missed diagnoses. Barriers to testing should be addressed to enable proper medical supervision. The suitability of testing frequencies to PrEP users with less frequent risk exposures needs to be evaluated.

## Introduction

Randomised controlled trials and observational studies have proven tenofovir disoproxil/emtricitabine (TDF/FTC) as HIV pre-exposure prophylaxis (PrEP) to be effective in preventing HIV infection [1-5]. To ensure safe use, medical supervision before and during PrEP use is recommended. Before initiating PrEP, users should be screened for HIV, hepatitis B virus (HBV) and impaired kidney function [6-8]. During PrEP use, the users should test for HIV at least every 3 months to confirm their HIV-negative status [6]. Kidney function should also be monitored regularly, since TDF can impair renal function [9-11]. Since PrEP users might be at an increased risk of sexually transmitted infections (STI) [12,13], guidelines recommend testing for syphilis every 3 months and for gonorrhoea and chlamydia every 3–6 months [6,7]. Regular screening may increase detection of asymptomatic STI and reduce their spread across sexual networks, although direct evidence for this is still lacking [14,15]. While all tests can be taken in medical practices in Germany, some can also be taken at anonymous testing sites (HIV and STI) or using self-tests (HIV) [16-18].

Few studies have investigated testing frequencies outside structured PrEP programmes. Data from surveys and insurance claims indicate considerable differences between guideline recommendations and actual testing frequencies [19-23]. Using PrEP from informal sources (i.e. unofficial sources like Internet platforms, dealers or friends) was associated with an overall lack of testing before and during PrEP use as well as lower testing frequencies among the ones who obtained any testing during PrEP use [23,24].

In Germany, PrEP is recommended for people with a 'substantial' risk of HIV infection [6]. Before September 2019, PrEP users in Germany had to cover the costs for generic PrEP (EUR 40–70 per month) and medical tests (up to EUR 100 per round of tests) themselves. Since then, these costs have been covered by statutory health insurances [25].

There is a lack of published data on testing frequencies and associated factors during PrEP use in a setting of self-funded PrEP. Insufficient testing frequencies among PrEP users may lead to missed or delayed diagnoses and potentially onward transmission of HIV and STI. Here, we investigated these outcomes by recruiting current PrEP users in Germany between July and October 2018 and between April and June 2019, when individuals had to pay themselves for PrEP and for testing .

## Methods

### Study design

The PrApp study (HIV Pre-exposure prophylaxis use among men who have sex with men (MSM) using dating apps) is an anonymous cross-sectional study investigating PrEP use in Germany [24]. Participants were recruited between 24 July and 30 October 2018 (Wave 1) and between 1 April and 15 June 2019 (Wave 2) via MSM dating apps (Grindr, Romeo, Hornet) in Germany, a German PrEP community website (https://prepjetzt.de) and German anonymous HIV/STI testing sites. Moreover, participants were asked to recruit friends. After providing consent, PrEP users completed an anonymous online survey on PrEP use, testing behaviour, and STI history, which was offered in German, Arabic, Dutch, English, French, Polish, Russian, Spanish and Turkish.

### Participant selection

We included all participants 18 years and older who provided consent to participate in the survey and were current PrEP users (i.e. everyone who self-reported to currently take PrEP). While the study was targeted to MSM, anyone was allowed to participate. We excluded participants who indicated current PrEP use while being HIV-positive and under medical supervision. To avoid including the same participant twice in our dataset, we excluded participants from Wave 2 of the study if they answered 'Yes' to the question if they had already participated in Wave 1.

### Outcomes and covariables

The outcomes of this analysis were (i) self-reported testing frequency for HIV, STI and renal function and (ii) self-reported STI history.

The survey questions can be found in the Supplement, section S11. Where not otherwise specified, the corresponding variables were grouped as shown in the tables. In accordance with German guideline recommendations [6], the frequency of HIV testing during current PrEP use was categorised as follows: every 3 months or more often, less often than every 3 months, or not at all. The frequency of STI testing was categorised as: every 6 months or more often, less often than every 6 months, or not at all. For the frequency of renal function testing, we used recommendations from the World Health Organization [11] because the German guideline required information on kidney function, which was not measured here. Kidney function testing was categorised into: every 3 months or more often, every 3–12 months, less often than annually, or not at all. Participants not receiving tests before initiating or during current PrEP use could indicate possible reasons. We defined any HIV testing frequency less often than every 3 months and any STI testing frequency less often than every 6 months as not consistent with German guidelines [6]. Renal function testing frequency less often than every 3 months was considered insufficient for participants in their first year of PrEP use and less than annually was considered insufficient for participants who had used PrEP for longer than 1 year [11].

We grouped gender as cisgender male if the gender identity and sex assigned at birth (where available) were male, cisgender female if the gender identity and sex assigned at birth (where available) were female. Gender-diverse included anyone indicating being transgender, non-binary or intersex. We grouped source of current PrEP into 'medical prescription' and 'informal' (as described in [24]). Anyone indicating not paying for tests was categorised as 'cost coverage', while participants indicating paying any amount for testing were categorised as 'self-payment'. The testing locations were categorised as 'testing only at the physician's', 'testing at the physician's and other locations', and 'only using checkpoints, self-tests or other locations'. Checkpoints are anonymous testing sites in Germany.

Self-reported STI history was recorded as having ever been diagnosed with syphilis, gonorrhoea, chlamydia, hepatitis B or hepatitis C. Using information about time since starting PrEP and time since last STI diagnosis, the last STI diagnosis was classified either as 'during PrEP use' or 'before PrEP use'.

### Statistical analysis

We analysed continuous variables using medians and interquartile ranges (IQR) and categorical variables using proportions and chi-squared tests where appropriate.

Factors associated with testing behaviour not consistent with guidelines were identified using univariable logistic regression models. In addition, we constructed a multivariable model to estimate the adjusted effect of informal current PrEP use on inconsistent testing behaviour. We included age and gender as forced variables in the model. Using a directed acyclic graph, we identified annual gross income, country of origin and type of current PrEP use as additional confounders (Supplement, section S1 shows the DAG and describes the identification of confounders). The p values from the regression models were derived using Wald tests. Patients with missing data for any of the variables in the multivariable model were excluded from the multivariable analysis. To assess the representativeness of the included study population, we compared participants who were in- and excluded from the multivariable analyses.

### Sensitivity analysis

In sensitivity analyses, we stratified the regression analyses by study waves in order to assess if the reported effects were similar between Wave 1 and Wave 2. In addition, we excluded participants who received PrEP through a clinical trial because their testing behaviour might differ from those receiving PrEP through routine medical care.

### Ethical statement

This study was approved by the ethics commission of the Berlin Chamber of Physicians (Ref: Eth-14/18). Participants actively provided informed consent for study participation.

## Results

We recruited 2,337 current and former PrEP users in Wave 1 of the survey and 3,484 current and former PrEP users in Wave 2 (Supplement, section S2 provides an overview on the selection of participants). We excluded 364 participants from Wave 2 because they had previously participated in Wave 1. After exclusion of 609 former PrEP users, we arrived at a final sample of 4,848 current PrEP users (Wave 1: 2,118, Wave 2: 2,730, Table 1). The median age was 37 years (IQR: 30–45). Overall, 88.7% were male, 1.3% were gender-diverse, and two participants were female. Because of the small sample size, female participants were not analysed as an independent group.

**Table 1:** Baseline data and testing frequency in current PrEP users, Germany, 2018 and 2019 (n = 4,848)

	Wave 1 (2018) N = 2,118		Wave 2 (2019) N = 2,730		Total N = 4,848	
	n	%	n	%	n	%
Age (years)						
Median (IQR)	38 (31-45)		36 (30-45)		37 (30-45)	
18–29	341	16.1	626	22.9	967	19.9
30–39	637	30.1	983	36.0	1,620	33.4
40–49	519	24.5	680	24.9	1,199	24.7
50–80	232	11.0	365	13.4	597	12.3
Missing	389	18.4	76	2.8	465	9.6
Gender						
Cisgender male	1,712	80.8	2,587	94.8	4,299	88.7
Cisgender female	0	0.0	2	0.1	2	0.0
Gender-diverse	16	0.8	48	1.8	64	1.3
Missing	390	18.4	93	3.4	483	10.0
Self-reported STI diagnoses during current PrEP use						
Syphilis	197	9.3	254	9.3	451	9.3
Gonorrhoea	340	16.1	422	15.5	762	15.7
Chlamydia	341	16.1	401	14.7	742	15.3
Hepatitis B	11	0.5	23	0.8	34	0.7
Hepatitis C	19	0.9	17	0.6	36	0.7
Missing	563	26.6	825	30.2	1,388	28.6
Obtained testing before starting PrEP						
Yes	1,785	84.3	2,096	76.8	3,881	80.1
No	73	3.4	93	3.4	166	3.4
Missing	260	12.3	541	19.8	801	16.5
Obtained testing during current PrEP use						
Yes	1,595	75.3	1,920	70.3	3,515	72.5
No	201	9.5	189	6.9	390	8.0
Missing	322	15.2	621	22.7	943	19.5
Frequency of HIV testing						
At least every 3 months	1,328	62.7	1,541	56.4	2,869	59.2
Less than every 3 months	245	11.6	375	13.7	620	12.8
Not at all	202	9.5	201	7.4	403	8.3
Missing	343	16.2	613	22.5	956	19.7
Frequency of STI testing						
At least every 6 months	1,391	65.7	1,611	59.0	3,002	61.9
Less than every 6 months	118	5.6	246	9.0	364	7.5
Not at all	218	10.3	212	7.8	430	8.9
Missing	391	18.5	661	24.2	1,052	21.7
Frequency of renal function testing						
At least every 3 months	1,082	51.1	1,230	45.1	2,312	47.7
Every 3–12 months	336	15.9	448	16.4	784	16.2
Less than annually	17	0.8	20	0.7	37	0.8
Not at all	243	11.5	268	9.8	511	10.5
Missing	440	20.8	764	28.0	1,204	24.8

HIV: human immunodeficiency virus; IQR: interquartile range; PrEP: pre-exposure prophylaxis; STI: sexually transmitted infection.

We obtained information on STI history and the timing of their last diagnosis from 3,460 participants (Table 1). Excluding participants with missing data, 22.0% (762/3,460) had received at least one gonorrhoea diagnosis, 21.4% (742/3,460) had received at least one chlamydia diagnosis and 13.0% (451/3,460) had received at least one syphilis diagnosis while using PrEP.

Among all participants, 3.4% indicated not receiving any medical tests (e.g. for HIV or STI) before starting PrEP and 8.0% were not getting tested during current PrEP use (Table 1). Among those, the most common reason for not getting tested before starting PrEP was not wanting to take the tests (33.3%; 31/93) while the most common reason for not getting tested during PrEP use was not knowing having to take the tests (33.3%; 63/189) (see Supplement, section S3 for a detailed list of these responses).

Most participants received HIV and renal function testing at least every 3 months and STI testing at least every 6 months (Table 1). Disregarding participants with missing data and accounting for duration of PrEP use for renal function testing frequencies, the testing frequency was less frequent than recommended for 26.3% regarding HIV tests, 20.9% regarding STI tests and 29.2% regarding renal function tests. The proportions were comparable between the study waves (see Supplement, section S4–S6 for a detailed breakdown of these data by study waves).

The proportion of PrEP users with at least one syphilis, gonorrhoea or chlamydia diagnosis during PrEP use was significantly higher in participants with guideline-recommended STI testing frequencies than in participants with lower STI testing frequencies (Table 2). This finding was numerically consistent across all substrata of sexual partner numbers and condom use. The only exception to this was the proportion of participants with at least one syphilis diagnosis during PrEP use, where we found comparable proportions across strata of condom use.

**Table 2:** History of sexually transmitted diseases during current PrEP use, stratified by testing frequency, Germany, 2018 and 2019 (n = 2,203)

	PrEP users with adequate STI testing frequencies (n = 1,997)			PrEP users with inadequate STI testing frequencies (n = 206)			p value^a^
	%	n	N	%	n	N	
PrEP users with at least one diagnosis of syphilis during current PrEP use							
Overall	20.9	417	1,997	12.6	26	206	0.005
By number of anal/vaginal sex partners within the last 6 months^b^							
0–3	18.1	28	155	11.5	3	26	0.414
4–10	17.1	98	574	9.4	6	64	0.114
> 10	22.8	281	1,231	14.5	16	110	0.045
By condom use while taking PrEP^c^							
Always/often	11.9	38	319	11.4	5	44	0.916
Inconsistent	22.7	377	1,662	12.2	19	156	0.002
PrEP users with at least one diagnosis of gonorrhoea during current PrEP use							
Overall	35.4	706	1,997	22.3	46	206	< 0.001
By number of anal/vaginal sex partners within the last 6 months^b^							
0–3	20.6	32	155	15.4	4	26	0.534
4–10	27.5	158	574	20.3	13	64	0.217
> 10	40.6	500	1,231	25.5	28	110	0.002
By condom use while taking PrEP^c^							
Always/often	20.7	66	319	15.9	7	44	0.458
Inconsistent	38.2	635	1,662	23.7	37	156	< 0.001
PrEP users with at least one diagnosis of chlamydia during current PrEP use							
Overall	34.5	689	1,997	21.8	45	206	< 0.001
By number of anal/vaginal sex partners within the last 6 months^b^							
0–3	14.8	23	155	3.8	1	26	0.126
4–10	27.0	155	574	17.2	11	64	0.090
> 10	40.5	499	1,231	30.0	33	110	0.030
By condom use while taking PrEP^c^							
Always/often	21.6	69	319	15.9	7	44	0.382
Inconsistent	37.0	615	1,662	24.4	38	156	0.002

PrEP: pre-exposure prophylaxis; STI: sexually transmitted infection.

^a^ Chi-squared test.

^b^ Excluding 43 participants with missing data on partner numbers.

^c^ Excluding 22 participants with missing data on partner numbers.

The strongest factor associated with inadequate HIV testing frequencies in the univariable analyses was not obtaining any testing before starting PrEP (OR = 18.0; 95% CI: 11.5–28.3; p < 0.001), followed by on-demand or intermittent PrEP use (OR = 7.3; 95% CI: 6.2–8.6; p < 0.001) and obtaining PrEP from informal sources (OR = 5.3; 95% CI: 4.4–6.4; p < 0.001) (Table 3). Other associated factors included being born outside Germany, having an annual gross income less than EUR 30,000, testing only at checkpoints, self-tests or other locations, having 10 or fewer sex partners in the last 6 months and always/often using condoms. While we also found some evidence that participants aged 18–29 years (OR = 1.3; 95% CI: 1.1–1.6; p = 0.004) or gender-diverse participants (OR = 1.7; 95% CI: 1.0–3.1: p = 0.051) were more likely to have inadequate testing frequencies; this was only apparent in Wave 1 of the study and not in Wave 2 (Supplement, section S4 provides an analysis of the regression models stratified by study waves).

**Table 3:** Factors associated with HIV testing behaviour less frequent than recommended by guidelines among current PrEP users, Germany, 2018 and 2019 (n = 3,892)

	Adequate HIV testing frequency n = 2,869		Inadequate HIV testing frequency n = 1,023		Univariable analysis^a^		Multivariable analysis^b^	
					OR (95% CI)	p value^c^	aOR (95% CI)	p value^c^
Source of current PrEP								
Medical prescription	2,593	90.4	662	64.7	1		1	
Informal	265	9.2	359	35.1	5.3 (4.4–6.4)	< 0.001	3.6 (2.9–4.5)	< 0.001
Missing	11	0.4	2	0.2	Not included			
Type of current PrEP use								
Daily	2,318	80.8	381	37.2	1		1	
On demand/intermittent	514	17.9	616	60.2	7.3 (6.2–8.6)	< 0.001	6.0 (5.0–7.2)	< 0.001
Missing	37	1.3	26	2.5	Not included			
Age (years)								
18–29	555	19.3	240	23.5	1.3 (1.1–1.6)	0.004	1.2 (0.9–1.5)	0.141
30–39	1,057	36.8	343	33.5	1		1	
40–49	827	28.8	254	24.8	0.9 (0.8–1.1)	0.562	0.8 (0.6–1.0)	0.048
50–80	378	13.2	149	14.6	1.2 (1.0–1.5)	0.091	1.0 (0.8–1.4)	0.913
Missing	52	1.8	37	3.6	Not included			
Country of origin								
Germany	1,892	65.9	600	58.7	1		1	
Outside Germany	560	19.5	244	23.9	1.4 (1.2–1.6)	< 0.001	1.2 (0.9–1.4)	0.152
Missing	417	14.5	179	17.5	Not included			
Annual gross income (EUR)								
< 30,000	711	24.8	281	27.5	1.3 (1.1–1.6)	0.005	1.1 (0.8–1.4)	0.536
30,000–49,000	840	29.3	251	24.5	1		1	
≥ 50,000	1,050	36.6	372	36.4	1.2 (1.0–1.4)	0.070	1.3 (1.0–1.6)	0.024
Missing	268	9.3	119	11.6	Not included			
Gender								
Cisgender male	2,782	97.0	966	94.4	1		1	
Gender-diverse	33	1.2	20	2.0	1.7 (1.0–3.1)	0.051	0.9 (0.5–2.0)	0.891
Missing	54	1.9	37	3.6	Not included			
Test before starting PrEP								
Yes	2,844	99.1	877	85.7	1		^d^	
No	23	0.8	128	12.5	18.0 (11.5–28.3)	< 0.001		
Missing	2	0.1	18	1.8	Not included			
Payment for testing								
Cost coverage^e^	1,556	54.2	324	31.7	1		^d^	
Self-payment	1,117	38.9	239	23.4	1.0 (0.9–1.2)	0.772		
Missing	196	6.8	460	45.0	Not included			
Location of testing								
Physician	2,151	75.0	394	38.5	1		^d^	
Physician and other locations	322	11.2	62	6.1	1.1 (0.8–1.4)	0.738		
Only using checkpoints, self-tests or other locations	308	10.7	142	13.9	2.5 (2.0–3.2)	< 0.001		
Missing	88	3.1	425	41.5	Not included			
Number of anal/vaginal sex partners within the last 6 months								
0–3	327	11.4	217	21.2	2.7 (2.2–3.3)	< 0.001	^d^	
4–10	891	31.1	372	36.4	1.7 (1.4–2.0)	< 0.001		
> 10	1,561	54.4	385	37.6	1			
Missing	90	3.1	49	4.8	Not included			
Condom use while taking PrEP								
Always/often	551	19.2	256	25.0	1.4 (1.2–1.7)	< 0.001	^d^	
About half of the times/sometimes/never	2,251	78.5	730	71.4	1			
Missing	67	2.3	37	3.6	Not included			

aOR: adjusted odds ratio; CI: confidence interval; HIV: human immunodeficiency virus; OR: odds ratio; PrEP: pre-exposure prophylaxis.

^a^ Univariable logistic regression model.

^b^ Multivariable logistic regression model to investigate the association of using PrEP from informal sources and infrequent testing behaviour including 2,338 participants with adequate and 808 participants with inadequate HIV test frequency, adjusting for age, gender, country of origin, annual gross income and type of PrEP use.

^c^ Wald test.

^d^ Not included in the multivariable regression model (see Supplement, section S1 for selection of confounders).

^e^ For some PrEP users, the costs for tests can be covered by health insurance in case of symptomatic infection or HIV/STI diagnoses among sexual partners or through clinical trials.

The factors associated with less than recommended STI and renal function testing frequencies in the univariable analyses were the same as the ones associated with infrequent HIV testing (Tables 4 and 5). Moreover, having to pay for the tests was associated with less frequent STI testing (OR = 1.4; 95% CI: 1.1–1.7; p = 0.006), but not with less frequent HIV or renal function testing (Tables 3 and 5). In addition, testing at the physician's and at other locations was associated with infrequent renal function testing compared with testing only at the physician's (Table 5). However, this association was not observed with infrequent HIV and STI testing (Tables 3 and 4).

**Table 4:** Factors associated with STI testing behaviour less frequent than recommended by guidelines, among current PrEP users, Germany, 2018 and 2019 (n = 3,796)

	Adequate STI test frequency n = 3,002		Inadequate STI test frequency n = 794		Univariable analysis^a^		Multivariable analysis^b^	
					OR (95% CI)	p value^c^	aOR (95% CI)	p value^c^
Source of current PrEP								
Medical prescription	2,650	88.3	521	65.6	1		1	
Informal	340	11.3	272	34.3	4.1 (3.4–4.9)	< 0.001	2.7 (2.1–3.3)	< 0.001
Missing	12	0.4	1	0.1	Not included			
Type of current PrEP use								
Daily	2,315	77.1	324	40.8	1		1	
On demand/intermittent	651	21.7	457	57.6	5.0 (4.2–5.9)	< 0.001	4.2 (3.5–5.1)	< 0.001
Missing	36	1.2	13	1.6	Not included			
Age (years)								
18–29	586	19.5	194	24.4	1.4 (1.1–1.7)	0.001	1.4 (1.1–1.8)	0.013
30–39	1,115	37.1	261	32.9	1		1	
40–49	850	28.3	209	26.3	1.1 (0.9–1.3)	0.634	1.0 (0.8–1.2)	0.847
50–80	398	13.3	109	13.7	1.2 (0.9–1.5)	0.220	1.0 (0.8–1.4)	0.754
Missing	53	1.8	21	2.6	Not included			
Country of origin								
Germany	1,961	65.3	480	60.5	1		1	
Outside Germany	604	20.1	192	24.2	1.3 (1.1–1.6)	0.007	1.1 (0.9–1.4)	0.361
Missing	437	14.6	122	15.4	Not included			
Annual gross income (EUR)								
< 30,000	757	25.2	221	27.8	1.3 (1.0–1.6)	0.031	1.0 (0.8–1.3)	0.957
30,000–49,000	863	28.7	199	25.1	1		1	
≥ 50,000	1,117	37.2	284	35.8	1.1 (0.9–1.3)	0.343	1.1 (0.9–1.4)	0.400
Missing	265	8.8	90	11.3	Not included			
Gender								
Cisgender male	2,906	96.8	764	96.2	1		1	
Gender-diverse	41	1.4	9	1.1	0.8 (0.4–1.7)	0.626	0.4 (0.2–1.0)	0.057
Missing	55	1.8	21	2.6	Not included			
Test before starting PrEP								
Yes	2,975	99.1	652	82.1	1		^d^	
No	23	0.8	127	16.0	25.2 (16.0–39.6)	< 0.001		
Missing	4	0.1	15	1.9	Not included			
Payment for testing								
Cost coverage^e^	1,663	55.4	185	23.3	1		^d^	
Self-payment	1,150	38.3	174	21.9	1.4 (1.1–1.7)	0.006		
Missing	189	6.3	435	54.8	Not included			
Location of testing								
Physician	2,208	73.6	275	34.6	1		^d^	
Physician and other locations	335	11.2	42	5.3	1.0 (0.7–1.4)	0.970		
Only using checkpoints, self-tests or other locations	381	12.7	66	8.3	1.4 (1.0–1.9)	0.026		
Missing	78	2.6	411	51.8	Not included			
Number of anal/vaginal sex partners within the last 6 months								
0–3	367	12.2	154	19.4	2.0 (1.6–2.6)	< 0.001	^d^	
4–10	948	31.6	288	36.3	1.5 (1.2–1.8)	< 0.001		
> 10	1,591	53.0	326	41.1	1			
Missing	96	3.2	26	3.3	Not included			
Condom use while taking PrEP								
Always/often	583	19.4	202	25.4	1.4 (1.2–1.7)	< 0.001	^d^	
About half of the times/sometimes/never	2,355	78.4	568	71.5	1			
Missing	64	2.1	24	3.0	Not included			

aOR: adjusted odds ratio; CI: confidence interval; OR: odds ratio; PrEP: pre-exposure prophylaxis; STI: sexually transmitted infection.

^a^ Univariable logistic regression model.

^b^ Multivariable logistic regression model to investigate the association of informal PrEP use and infrequent testing behaviour including 2,452 participants with adequate and 639 participants with inadequate STI test frequency, adjusting for age, gender, country of origin, annual gross income and type of PrEP use.

^c^ Wald test.

^d^ Not included in the multivariable regression model (see Supplement, section S1 for selection of confounders).

^e^ For some PrEP users, the costs for tests can be covered by health insurance in case of symptomatic infection or HIV/STI diagnoses among sexual partners or through clinical trials.

**Table 5:** Factors associated with renal function testing behaviour less frequent than recommended by guidelines, among current PrEP users, Germany, 2018 and 2019 (n = 3,618)

	Adequate renal test frequency n = 2,561		Inadequate renal test frequency n = 1,057		Univariable analysis^a^		Multivariable analysis^b^	
					OR (95% CI)	p value^c^	aOR (95% CI)	p value^c^
Source of current PrEP								
Medical prescription	2,316	90.4	714	67.5	1		1	
Informal	235	9.2	340	32.2	4.7 (3.9–5.7)	< 0.001	2.9 (2.3–3.7)	< 0.001
Missing	10	0.4	3	0.3	Not included			
Type of current PrEP use								
Daily	2,103	82.1	458	43.3	1		1	
On demand/intermittent	452	17.6	597	56.5	6.1 (5.2–7.1)	< 0.001	4.9 (4.1–5.9)	< 0.001
Missing	6	0.2	2	0.2	Not included			
Age (years)								
18–29	475	18.5	266	25.2	1.4 (1.2–1.7)	< 0.001	1.4 (1.1–1.7)	0.011
30–39	946	36.9	374	35.4	1		1	
40–49	767	29.9	260	24.6	0.9 (0.7–1.0)	0.103	0.8 (0.6–1.0)	0.043
50–80	359	14.0	142	13.4	1.0 (0.8–1.3)	0.997	0.9 (0.7–1.2)	0.403
Missing	14	0.5	15	1.4	Not included			
Country of origin								
Germany	1,774	69.3	627	59.3	1		1	
Outside Germany	487	19.0	266	25.2	1.5 (1.3–1.8)	< 0.001	1.3 (1.1–1.6)	0.006
Missing	300	11.7	164	15.5	Not included			
Annual gross income (EUR)								
< 30,000	633	24.7	302	28.6	1.2 (1.0–1.5)	0.025	1.0 (0.8–1.2)	0.790
30,000–49,000	760	29.7	291	27.5	1		1	
≥ 50,000	997	38.9	378	35.8	1.0 (0.8–1.2)	0.914	1.0 (0.8–1.2)	0.774
Missing	171	6.7	86	8.1	Not included			
Gender								
Cisgender male	2,516	98.2	1,025	97.0	1		1	
Gender-diverse	29	1.1	17	1.6	1.4 (0.8–2.6)	0.237	0.9 (0.4–2.0)	0.845
Missing	16	0.6	15	1.4	Not included			
Test before starting PrEP								
Yes	2,545	99.4	914	86.5	1		^d^	
No	14	0.5	127	12.0	25.3 (14.5–44.1)	< 0.001		
Missing	2	0.1	16	1.5	Not included			
Payment for testing								
Cost coverage^e^	1,430	55.8	351	33.2	1		^d^	
Self-payment	1,028	40.1	272	25.7	1.1 (0.9–1.3)	0.407		
Missing	103	4.0	434	41.1	Not included			
Location of testing,								
Physician	2,002	78.2	413	39.1	1		^d^	
Physician and other locations	282	11.0	86	8.1	1.5 (1.1–1.9)	0.004		
Only using checkpoints, self-tests, or other locations	267	10.4	161	15.2	2.9 (2.3–3.7)	< 0.001		
Missing	10	0.4	397	37.6	Not included			
Number of anal/vaginal sex partners within the last 6 months								
0–3	302	11.8	204	19.3	2.2 (1.8–2.7)	< 0.001	^d^	
4–10	796	31.1	399	37.7	1.6 (1.4–1.9)	< 0.001		
> 10	1,412	55.1	430	40.7	1			
Missing	51	2.0	24	2.3	Not included			
Condom use while taking PrEP								
Always/often	485	18.9	265	25.1	1.4 (1.2–1.7)	< 0.001	^d^	
About half of the times/sometimes/never	2,049	80.0	779	73.7	1			
Missing	27	1.1	13	1.2	Not included			

CI: confidence interval; OR: odds ratio; PrEP: pre-exposure prophylaxis; STI: sexually transmitted infection.

^a^ Univariable logistic regression model.

^b^ Multivariable logistic regression model to investigate the association of informal PrEP use and infrequent testing behaviour including 2,155 participants with adequate and 858 participants with inadequate renal testing frequency, adjusting for age, gender, country of origin, annual gross income and type of PrEP use.

^c^ Wald test.

^d^ Not included in the multivariable regression model (see Supplement, section S1 for selection of confounders).

^e^ For some PrEP users, the costs for tests can be covered by health insurance in case of symptomatic infection or HIV/STI diagnoses among sexual partners or through clinical trials.

In the multivariable model investigating the influence of obtaining PrEP from informal sources on testing frequencies, we found a positive effect on inadequate HIV testing frequencies (adjusted OR = 3.6; 95% CI: 2.9–4.5; p < 0.001), STI testing frequencies (adjusted OR = 2.7; 95% CI: 2.1–3.3; p < 0.001), and renal function testing frequencies (adjusted OR = 2.9; 95% CI: 2.3–3.7; p < 0.001). Participants in- and excluded from the multivariable analysis were comparable (Supplement, part S7 contains a comparison of participants in- and excluded in the regression models).

Apart from the different effects regarding age and gender identity described above, the other effects were comparable between the two study waves (see Supplement, section S4–S6 for regression analyses stratified by study waves). Sensitivity analyses excluding participants who obtained PrEP through a clinical trial yielded similar results (see Supplement, section S8–S10 for regression analyses excluding participants who obtained PrEP through a clinical trial).

## Discussion

We investigated the testing frequencies for HIV, STI and renal function and the self-reported STI diagnoses in a large sample of PrEP users recruited in 2018 and 2019 in Germany when PrEP and medical testing were not covered by health insurance. A distinct proportion of the participants reported testing frequencies during PrEP use less often than recommended by German guidelines for HIV (26.3%), STI (20.9%) and renal function (29.2%). Participants with frequent STI testing had higher proportions of self-reported STI diagnoses during PrEP use, overall and across strata of partner numbers and condom use. The strongest factors associated with less frequent HIV, STI and renal function testing were not reporting having any testing before starting PrEP, obtaining PrEP from informal sources and on-demand or intermittent PrEP use.

The HIV and STI testing frequencies in our study are higher than results from two studies in the United States (US) [20,22]. In US guidelines, HIV testing is recommended every 3 months and bacterial STI testing every 3–6 months [7]. An analysis of insurance claims data from 2011 to 2015 found that after 6 months of PrEP, 38% tested for HIV, 49% for syphilis, 39% for chlamydia/gonorrhoea, and 37% for kidney function [20]. Another study examined test ordering in a network of 15 San Francisco public health primary care clinics from 2013 to 2017 [22]. Among PrEP users, HIV tests were ordered by providers in 68% of cases in 4-month intervals; STI testing was ordered in 67% of cases in 6-month intervals and creatinine testing was ordered in 71.3% of cases in 6-month intervals. The difference to our results might be due to differences in data capture and healthcare systems. A US survey showed that regular STI screening was offered to 57–87% of PrEP users depending on the sampling location [19]. In a survey from the United Kingdom, self-reported HIV/STI testing frequencies were high among PrEP users in a trial or a PrEP programme but lower for users sourcing PrEP privately (HIV: 58% reported ≥ 3 tests/year; STI: 48% reported ≥ 3 tests/year) [23]. In all these studies, testing frequencies were lower than recommended among some PrEP users, which necessitates a better understanding of the underlying factors to address these disparities.

Using PrEP from informal sources and not obtaining any tests before starting PrEP may reflect similar access barriers to obtaining appropriate medical supervision during PrEP use [24]. Since informal PrEP users can obtain PrEP through different sources, e.g. websites, travelling to other countries or friends, they may lack the knowledge on proper testing intervals, where to find testing facilities, or how to obtain testing through physicians. A qualitative study from the Netherlands showed that some informal PrEP users avoided renal function testing since they did not consider it necessary or had difficulties finding testing locations [26]. In addition, using PrEP from some informal sources (e.g. friends, cheaper Internet offers) may reflect financial constraints that also might make it difficult for people to obtain self-funded testing. We expect that the health insurance coverage in Germany since September 2019 has reduced barriers to obtain PrEP through medical care. However, stigma around PrEP might still prevent some people from seeking appropriate medical care and might keep them from obtaining regular testing. We will investigate barriers to regular testing in future surveys.

PrEP users with consistent condom use and low partner numbers may perceive themselves at a lower risk for contracting HIV and STI and thus seek testing less often. While the risk for HIV among people using PrEP as prescribed is already strongly reduced [2], regular HIV testing is still recommended to ensure that PrEP users do not have an undiagnosed HIV infection and to prevent development of drug resistance and passing on the virus [6]. While STI risks decrease with consistent condom use, STI can still be transmitted under these circumstances [27-30]. Thus, regular screening appears advisable even in the context of consistent condom use. The link between insufficient renal function testing and lower partner numbers or consistent condom use appears less obvious. However, since HIV, STI and renal function testing are often performed at the same time, reduced testing for HIV and STI might also influence the frequency of renal function testing.

The use of on-demand or intermittent PrEP was also associated with less frequent testing in our study. On-demand or intermittent PrEP use may be associated with a lower number of sexual partners or fewer potential transmission events compared with daily PrEP use, which could lead to a lower risk perception. Alternatively, people could use on-demand or intermittent PrEP because of limited finances and hence also refrain from medical testing for financial reasons. While financial reasons for on-demand PrEP use and less frequent testing might be addressed by health insurance coverage, there is evidence that even in a setting of cost coverage for PrEP, there is still a substantial interest for on-demand and intermittent PrEP use, e.g. because of suspected side effects of PrEP [31]. Thus, developing risk assessments and testing guidelines for on-demand/intermittent PrEP users and those with lower risk exposures might help promote a more needs-based testing regimen.

A factor only associated with less frequent STI testing, but not less frequent HIV or renal function testing, was self-payment for the tests. The observation that STI tests (e.g. gonorrhoea and chlamydia) are often more expensive than HIV or renal function tests might provide an explanation. Thus, cost coverage can contribute to ensure proper medical supervision during PrEP use, which is in agreement with survey data from 2020 [32].

Another factor that might influence PrEP users to obtain medical testing less frequently than recommended is that PrEP users may interpret prior negative results to mean that they are at lower risk for future STI. In addition, STI can remain asymptomatic so that PrEP users may not perceive a necessity to obtain testing [33]. Since we did not measure these factors in our surveys, future studies should address this.

In our study, PrEP users frequently reported STI during PrEP use. The reliability of our self-reported data on routinely screened STI (e.g. gonorrhoea and chlamydia) is corroborated by the results from the German MSM Screening study [33]. In that study, the prevalence of gonorrhoea and chlamydia in PrEP users was 14.8% and 13.8%, respectively, which was of similar magnitude as the self-reported prevalence of STI occurring during PrEP use in our study.

Among participants with less frequent testing than recommended by guidelines, the proportions of participants being diagnosed with STI were lower than among frequent testers. While individual risk behaviour and/or risk perception might drive people with a higher likelihood of contracting STI to obtaining tests more frequently and thus constitute confounding by indication, the observed difference was numerically consistent among all strata of condom use and sexual partner numbers. It is theoretically possible that some PrEP users with many partners might achieve a low STI risk through additional measures and that the lower STI prevalence could reflect a lower risk in reality. However, it appears more likely that asymptomatic STI were overlooked among the PrEP users who are not testing frequently, and that the true STI incidence in this group is higher than reported. This is supported by another study where inconsistent condom use and higher partner numbers were positively associated with an increased risk of STI diagnoses among current PrEP users [34].

Modelling studies have shown that periodic STI testing among PrEP users might reduce the incidence of these STI over time [14,35], although real-world evidence is still lacking. If this assumption holds true, frequent STI testing would be advisable in order to avoid missing infections. However, the discussion is still ongoing regarding the benefits of frequent screening with treatment of asymptomatic infections and potential risks of antibiotic resistance development [15,36].

In many countries e.g. in Eastern Europe, Latin America and Asia, national health systems do currently not provide coverage for PrEP and associated HIV, STI and renal function testing. Self-funded PrEP will become the default mode of providing PrEP for MSM in these settings. Our study provides important insights on how well self-funded PrEP users in a western European country may adhere to guideline-recommended testing frequencies and the factors associated with infrequent testing.

Among the strengths of our study is the large sample size allowing for a comprehensive investigation of the testing behaviour and STI history of PrEP users in Germany with sufficient statistical power. We provided the survey in nine languages to allow recruitment of a comprehensive sample of PrEP users irrespective of their ability to speak German.

Several limitations need to be considered. Most participants were recruited through MSM dating apps or an online website and they were mostly middle-aged so the study population may not be representative of all PrEP users in Germany and the generalisability to other PrEP users is limited. We were unable to collect information on non-participation so we could not investigate selection bias. The amount of missing data in some variables was considerable (e.g. self-reported STI diagnoses), which may also have influenced the results. Participants receiving PrEP through a clinical trial might be under stricter medical supervision and have higher testing frequencies. However, our sensitivity analysis excluding these participants yielded similar results. Since the study results are based on self-reported information, participants may have provided more socially acceptable answers on testing frequencies, type of PrEP use and sexual behaviour. While this might have led to an overestimation of the true testing frequencies, it would have made our groups for the regression analyses more alike and the effect estimates might be underestimated. However, the questionnaire was designed for anonymous participation and participants indicated risky sexual behaviour, informal PrEP use and high STI prevalence. Thus, we do not expect this to have a major effect. Furthermore, the answers provided in the study may be subject to recall bias and participants may not have been able to accurately recall their testing frequencies, number of partners or condom use. In addition, the analysed behaviours may also have changed over time, which would not be reflected in the dataset. Moreover, some participants may not have been aware that renal function tests had been done if they were ordered by their physicians as part of their standard bloodwork, and these participants may have been misclassified as less frequent renal function testers. Thus, the testing frequency for the renal function tests may be an underestimate. Since these effects are expected to affect all participants non-differentially, this would have made the groups more comparable and biased the results towards the null hypothesis. In addition, our findings regarding age and gender differed between the two study waves so that we cannot derive a consistent conclusion regarding the association between these factors and inconsistent testing frequencies.

## Conclusion

In 2018 and 2019, about one in four self-funded PrEP users in Germany obtained HIV testing less frequently than recommended, about one in five obtained STI testing less frequently than recommended and about one in three obtained renal function tests less frequently than recommended. Testing less frequently than recommended by guidelines can lead to missed diagnoses. We identified important factors for less frequent testing during PrEP use including not testing before starting PrEP, obtaining PrEP from informal sources and on-demand or intermittent PrEP use. While some barriers may be overcome by health insurance coverage of PrEP and the recommended medical tests, other factors indicate that some PrEP users have lower and/or more infrequent risks. Future research should investigate if the recommended testing frequencies are reasonable for this group of PrEP users.

## Supplementary Data

742000 Supplement
