# Supplementary material for: HIV, STI and renal function testing frequency and STI history among current users of self-funded HIV pre-exposure prophylaxis, a cross-sectional study, Germany, 2018 and 2019
Source: Euro Surveill. 2022 Apr 7;27(14):2100503. doi: 10.2807/1560-7917.ES.2022.27.14.2100503 (PMC8991737; doi:10.2807/1560-7917.ES.2022.27.14.2100503)
Supplement: Supplement [file 21-00503_KOPPE_Supplement.pdf]

Appendix: HIV, STI and renal function testing frequency and STI history among current users of HIV pre-exposure prophylaxis (PrEP) in a setting of self-funded PrEP, a cross-sectional study in Germany

This supplementary material is hosted by *Eurosurveillance* as supporting information alongside the article "HIV, STI and renal function testing frequency and STI history among current users of HIV pre-exposure prophylaxis (PrEP) in a setting of self-funded PrEP, a cross-sectional study in Germany", on behalf of the authors, who remain responsible for the accuracy and appropriateness of the content. The same standards for ethics, copyright, attributions and permissions as for the article apply. Supplements are not edited by *Eurosurveillance* and the journal is not responsible for the maintenance of any links or email addresses provided therein

Overview

Appendix S1: Directed acyclic graph to identify factors influencing the association between informal PrEP use and infrequent testing behaviour

Appendix S2: Participant selection for analysis

Appendix S3: Reasons for not getting tested before and during PrEP use

Appendix S4: Factors associated with HIV testing behaviour less frequent than recommended by guidelines stratified by study waves

Appendix S5: Factors associated with STI testing behaviour less frequent than recommended by guidelines stratified by study waves

Appendix S6: Factors associated with renal function testing behaviour less frequent than recommended by guidelines stratified by study waves

Appendix S7: Comparison of study participants with and without missing data

Appendix S 1: Factors associated with HIV testing behaviour less frequent than recommended by guidelines excluding participants receiving PrEP through a clinical trial

Appendix S 9: Factors associated with STI testing behaviour less frequent than recommended by guidelines excluding participants receiving PrEP through a clinical trial

Appendix S 10: Factors associated with renal function testing behaviour less frequent than recommended by guidelines excluding participants receiving PrEP through a clinical trial

Appendix S11: Survey questions.

Appendix S 1: Directed acyclic graph to identify factors influencing the association between informal PrEP use and infrequent testing behaviour

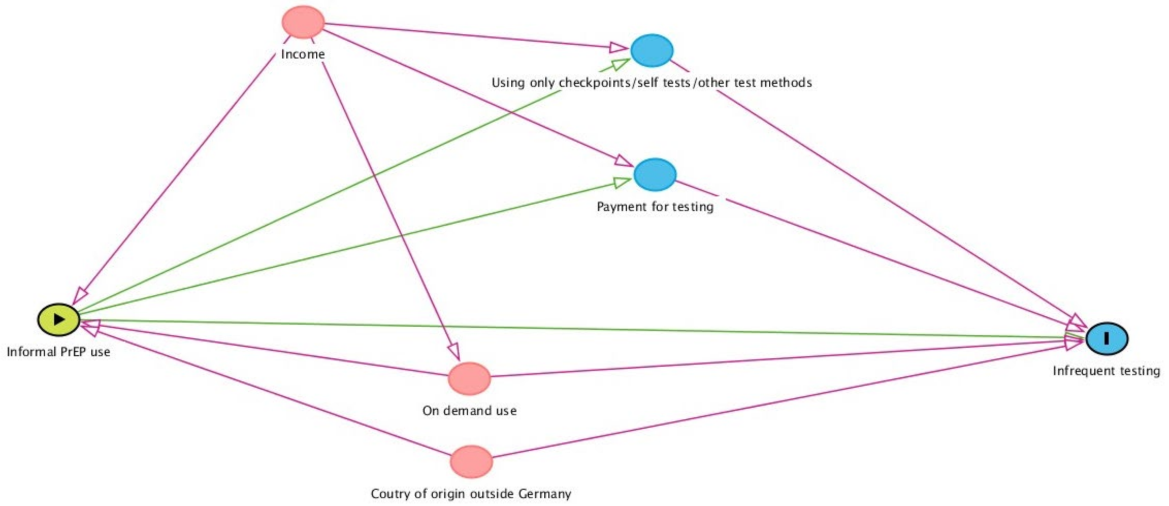

This directed acyclic graph was drawn to identify the adjusted effect of the exposure "informal PrEP use" on the outcome "infrequent testing behaviour".

Income was identified as a confounder, because in a setting of self-paid PrEP a lower income might influence people to use informal sources for PrEP. A lower income might also keep them from maintaining the guideline-recommended testing frequency since PrEP users had to pay for the tests themselves at the time of the study.

On demand PrEP users were more likely to use PrEP from informal sources in a previous analysis (Koppe et al, JIAS, 2019). Moreover, on demand PrEP use might also influence the testing frequency since people who do not want to use PrEP daily might also have a lower frequency of condomless sex encounters. Thus, on demand use was identified as a confounder.

People born outside Germany were more likely to use PrEP from informal sources in a previous analysis (Koppe et al, JIAS, 2019). In addition, they might also encounter barriers to access medical care due to language barriers or lack of health insurance coverage. Thus, being born outside Germany was identified as a confounder.

Other variables including partner numbers and type of condom use are not affecting the exposure (informal PrEP use) so they are not considered as confounding factors for this multivariable analysis. In addition, testing location and payment for testing are considered as mediator variables between informal PrEP use and infrequent testing behaviour. Since we want to estimate the total effect of informal PrEP use on infrequent testing behaviour, these variables are not included in the multivariable model.

Appendix S2: Participant selection for analysis of testing frequencies among current PrEP users in Germany, 2018-2019

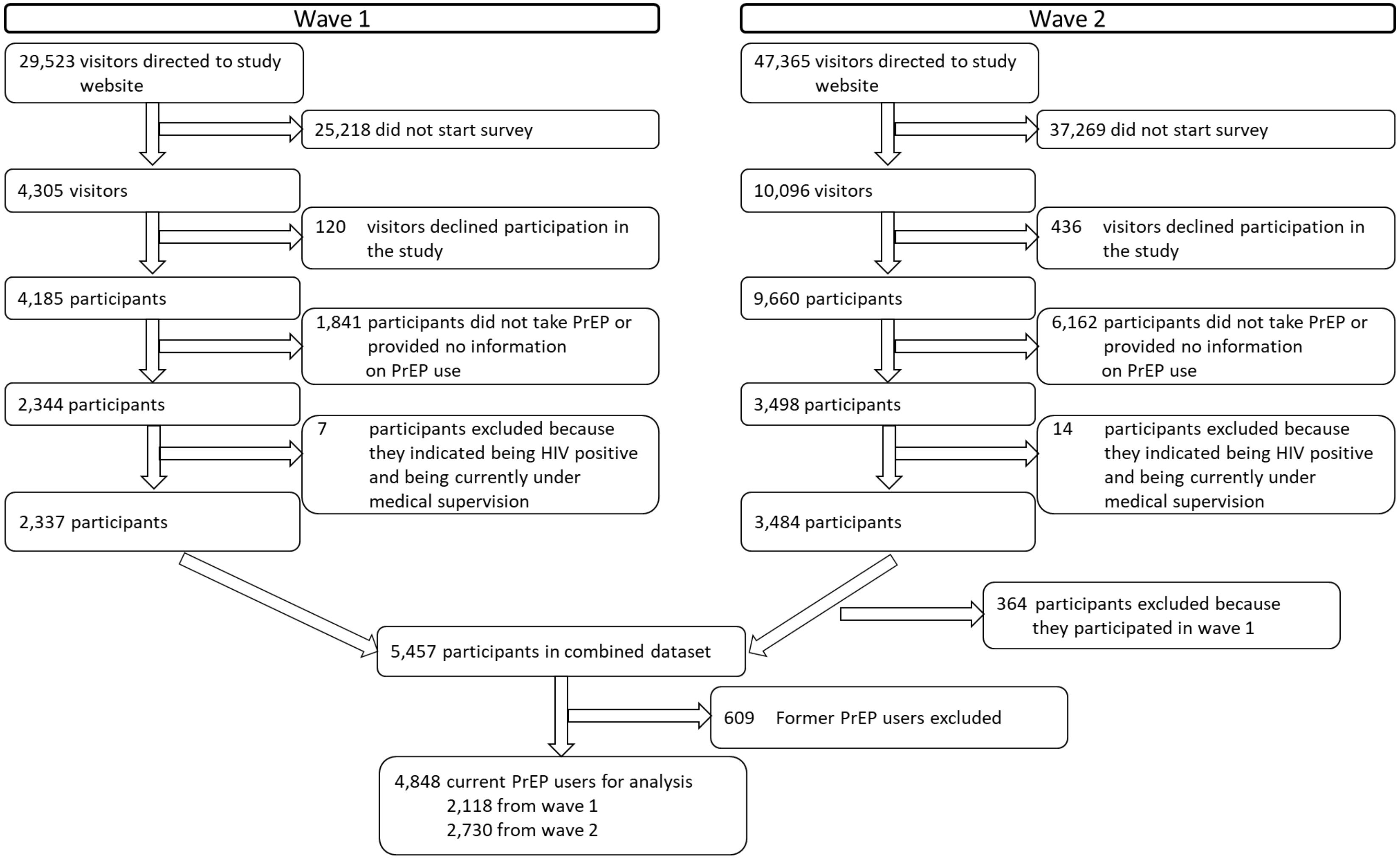

Appendix S3: Reasons for not getting tested before and during PrEP use among current PrEP users in Germany (only participants from study wave 2, 2019)

Reasons for no tests before PrEP use

Reason	Participants, n(%)
Cannot afford taking tests	16 (17.2%)
Tests were not offered	22 (23.7%)
Do not have time to take tests	22 (23.7%)
Do not want to take the tests	31 (33.3%)
Do not think I would benefit from taking tests	13 (14.0%)
Did not know that I needed to take tests	23 (24.7%)
Other reason	5 (5.4%)
Missing	2 (2.2%)
Total	93 (100%)

Reasons for no tests during PrEP use

Reason	Participants, n(%)
Cannot afford taking tests	24 (12.7%)
Tests were not offered	56 (29.6%)
Do not have time to take tests	33 (17.5%)
Do not want to take the tests	39 (20.6%)
Do not think I would benefit from taking tests	22 (11.6%)
Did not know that I needed to take tests	63 (33.3%)
Other reason	14 (7.4%)
Missing	8 (4.2%)
Total	189 (100%)

Appendix S4: Factors associated with HIV testing behaviour less frequent than recommended by guidelines among current PrEP users in Germany, 2018-2019; stratified by study waves

Wave 1 (n = 1,775)

	Adequate HIV test frequency	Inadequate HIV test frequency	Univariable Analysis[1]		Multivariable Analysis[2]	
			OR (95% CI)	p-value[3]	OR (95% CI)	p-value[3]
Total (n)	1328	447				
**Source of current PrEP, n(%)**						
Medical Prescription	1,172 (88.3%)	269 (60.2%)	1		1	
Informal	155 (11.7%)	176 (39.4%)	4.9 (3.8 – 6.4)	<0.001	3.6 (2.6 – 5.0)	<0.001
Missing	1 (0.1%)	2 (0.4%)	-			
**Type of current PrEP use, n(%)**						
Daily	1,084 (81.6%)	152 (34.0%)	1		1	
On demand / intermittent	209 (15.7%)	271 (60.6%)	9.2 (7.2 – 11.8)	<0.001	6.8 (5.1 – 9.0)	<0.001
Missing	35 (2.6%)	24 (5.4%)	-			
**Age (years)**						
18 – 29, n (%)	213 (16.0%)	117 (26.2%)	2.0 (1.5 – 2.7)	<0.001	1.6 (1.1 – 2.3)	0.025
30 – 39, n (%)	486 (36.6%)	135 (30.2%)	1		1	
40 – 49, n (%)	404 (30.4%)	103 (23.0%)	0.9 (0.7 – 1.2)	0.560	0.8 (0.5 – 1.1)	0.148
50 – 80, n (%)	173 (13.0%)	55 (12.3%)	1.1 (0.8 – 1.6)	0.460	1.0 (0.6 – 1.6)	0.991
Missing	52 (3.9%)	37 (8.3%)	-			
**Country of origin, n (%)**						
Germany	872 (65.7%)	257 (57.5%)	1		1	
Outside Germany	271 (20.4%)	104 (23.3%)	1.3 (1.0 – 1.7)	0.051	1.1 (0.8 – 1.5)	0.749
Missing	185 (13.9%)	86 (19.2%)	-			
**Annual gross income, n (%)**						
<30,000 €	290 (21.8%)	119 (26.6%)	1.5 (1.1 – 2.1)	0.004	1.1 (0.7 – 1.6)	0.731
30,000 – 49,000 €	419 (31.6%)	111 (24.8%)	1		1	
≥50,000 €	505 (38.0%)	154 (34.5%)	1.2 (0.9 – 1.5)	0.318	1.2 (0.8 – 1.7)	0.330
Missing	114 (8.6%)	63 (14.1%)				
**Gender, n (%)**						
Cisgender male	1,267 (95.4%)	403 (90.2%)	1		1	
Gender diverse	8 (0.6%)	7 (1.6%)	2.8 (1.0 – 7.6)	0.052	0.7 (0.2 – 2.7)	0.571
Missing	53 (4.0%)	37 (8.3%)	-			
**Test before starting PrEP, n(%)**						
No	5 (0.4%)	61 (13.6%)	1		[4]	
Yes	1,323 (99.6%)	377 (84.3%)	42.8 (17.1 – 107.3)	<0.001		
Missing	0 (0.0%)	9 (2.0%)	-			
**Payment for testing, n(%)**						
Cost coverage	693 (52.2%)	130 (29.1%)	1		[4]	
Self-payment	554 (41.7%)	83 (18.6%)	0.8 (0.6 – 1.1)	0.138		
Missing	81 (6.1%)	234 (52.3%)	-			
**Location of testing, n(%)**						
Physician	976 (73.5%)	138 (30.9%)	1		[4]	
Physician + other locations	161 (12.1%)	27 (6.0%)	1.2 (0.8 – 1.9)	0.452		
Only using checkpoints, self-tests, or other locations	161 (12.1%)	65 (14.5%)	2.9 (2.0 – 4.0)	<0.001		
Missing	30 (2.3%)	217 (48.5%)	-			
**Number of anal/vaginal sex partners within the last 6 months, n (%)**						
0 – 3	149 (11.2%)	87 (19.5%)	2.6 (1.9 – 3.5)	<0.001	[4]	
4 – 10	375 (28.2%)	152 (34.0%)	1.8 (1.4 – 2.3)	<0.001		
> 10	751 (56.6%)	170 (38.0%)	1			
Missing	53 (4.0%)	38 (8.5%)	-			
**Condom use while taking PrEP, n(%)**						
Always / Often	1,034 (77.9%)	316 (70.7%)	1.3 (1.0 – 1.7)	0.051	[4]	
In about half of the times/sometimes/never	246 (18.5%)	98 (21.9%)	1			
Missing	48 (3.6%)	33 (7.4%)	-			

[1] Univariable logistic regression model; [2] Multivariable logistic regression model to investigate the association of informal PrEP use and infrequent testing behaviour including 1,093 participants with adequate and 343 participants with inadequate HIV test frequency, adjusting for age, gender, country of origin, annual gross income, and type of PrEP use. [3] Wald test [4] Not included in the multivariable regression model (see appendix S1). CI: Confidence Interval, HIV: Human Immunodeficiency Virus, OR: Odds Ratio, PrEP: Pre-exposure prophylaxis

Wave 2 (n = 2,117)

	Adequate HIV test frequency	Inadequate HIV test frequency	Univariable Analysis[1]		Multivariable Analysis[2]	
			OR (95% CI)	p-value[3]	OR (95% CI)	p-value[3]
Total (n)	1541	576				
**Source of current PrEP, n(%)**						
Medical Prescription	1,421 (92.2%)	393 (68.2%)	1		1	
Informal	110 (7.1%)	183 (31.8%)	6.0 (4.6 – 7.8)	<0.001	4.0 (2.9 – 5.5)	<0.001
Missing	10 (0.6%)	0 (0.0%)				
**Type of current PrEP use, n(%)**						
Daily	1,234 (80.1%)	229 (39.8%)	1		1	
On demand / intermittent	305 (19.8%)	345 (59.9%)	6.1 (4.9 – 7.5)	<0.001	5.3 (4.1 – 6.8)	<0.001
Missing	2 (0.1%)	2 (0.3%)				
**Age (years)**						
18 – 29, n (%)	342 (25.8%)	123 (27.5%)	1.0 (0.8 – 1.3)	0.923	1.0 (0.7 – 1.4)	0.907
30 – 39, n (%)	571 (43.0%)	208 (46.5%)	1		1	
40 – 49, n (%)	423 (31.9%)	151 (33.8%)	1.0 (0.8 – 1.3)	0.871	0.8 (0.6 – 1.1)	0.201
50 – 80, n (%)	205 (15.4%)	94 (21.0%)	1.3 (0.9 – 1.7)	0.121	1.0 (0.7 – 1.5)	0.895
Missing	- -	- -				
**Country of origin, n (%)**						
Germany	1,020 (66.2%)	343 (59.5%)	1		1	
Outside Germany	289 (18.8%)	140 (24.3%)	1.4 (1.1 – 1.8)	0.002	1.3 (1.0 – 1.7)	0.104
Missing	232 (15.1%)	93 (16.1%)				
**Annual gross income, n (%)**						
<30,000 €	421 (27.3%)	162 (28.1%)	1.2 (0.9 – 1.5)	0.278	1.1 (0.8 – 1.5)	0.708
30,000 – 49,000 €	421 (27.3%)	140 (24.3%)	1		1	
≥50,000 €	545 (35.4%)	218 (37.8%)	1.2 (0.9 – 1.5)	0.143	1.4 (1.0 – 1.9)	0.036
Missing	154 (10.0%)	56 (9.7%)				
**Gender, n (%)**						
Cisgender male	1,515 (98.3%)	563 (97.7%)	1		1	
Gender diverse	25 (1.6%)	13 (2.3%)	1.4 (0.7 – 2.8)	0.331	1.1 (0.5 – 2.7)	0.839
Missing	1 (0.1%)	0 (0.0%)				
**Test before starting PrEP, n(%)**						
No	18 (1.2%)	67 (11.6%)	1		[4]	
Yes	1,521 (98.7%)	500 (86.8%)	11.3 (6.7 – 19.2)	<0.001		
Missing	2 (0.1%)	9 (1.6%)				
**Payment for testing, n(%)**						
Cost coverage	863 (56.0%)	194 (33.7%)	1		[4]	
Self-payment	563 (36.5%)	156 (27.1%)	1.2 (1.0 – 1.6)	0.082		
Missing	115 (7.5%)	226 (39.2%)				
**Location of testing, n(%)**						
Physician	1,175 (76.2%)	256 (44.4%)	1		[4]	
Physician + other locations	161 (10.4%)	35 (6.1%)	1.0 (0.7 – 1.5)	0.991		
Only using checkpoints, self-tests, or other locations	147 (9.5%)	77 (13.4%)	2.4 (1.8 – 3.3)	<0.001		
Missing	58 (3.8%)	208 (36.1%)				
**Number of anal/vaginal sex partners within the last 6 months, n (%)**						
0 – 3	178 (11.6%)	130 (22.6%)	2.8 (2.1 – 3.6)	<0.001	[4]	
4 – 10	516 (33.5%)	220 (38.2%)	1.6 (1.3 – 2.0)	<0.001		
> 10	810 (52.6%)	215 (37.3%)	1			
Missing	37 (2.4%)	11 (1.9%)				
**Condom use while taking PrEP, n(%)**						
Always / Often	1,217 (79.0%)	414 (71.9%)	1.5 (1.2 – 1.9)	<0.001	[4]	
In about half of the times/sometimes/never	305 (19.8%)	158 (27.4%)	1			
Missing	19 (1.2%)	4 (0.7%)				

[1] Univariable logistic regression model; [2] Multivariable logistic regression model to investigate the association of informal PrEP use and infrequent testing behaviour including 1,245 participants with adequate and 465 participants with inadequate HIV test frequency, adjusting for age, gender, country of origin, annual gross income, and type of PrEP use. [3] Wald test [4] Not included in the multivariable regression model (see appendix S1). CI: Confidence Interval, HIV: Human Immunodeficiency Virus, OR: Odds Ratio, PrEP: Pre-exposure prophylaxis

Appendix S5: Factors associated with STI testing behaviour less frequent than recommended by guidelines among current PrEP users in Germany, 2018-2019; stratified by study waves

Wave 1 (n = 1,727)

	Adequate STI test frequency	Inadequate STI test frequency	Univariable Analysis[1]		Multivariable Analysis[2]	
			OR (95% CI)	p-value[3]	OR (95% CI)	p-value[3]
Total (n)	1391	336				
**Source of current PrEP, n(%)**						
Medical Prescription	1,191 (85.6%)	208 (61.9%)	1		1	
Informal	197 (14.2%)	128 (38.1%)	3.7 (2.8 – 4.9)	<0.001	2.4 (1.7 – 3.3)	<0.001
Missing	3 (0.2%)	0 (0.0%)				
**Type of current PrEP use, n(%)**						
Daily	1,079 (77.6%)	130 (38.7%)	1		1	
On demand / intermittent	278 (20.0%)	195 (58.0%)	5.8 (4.5 – 7.5)	<0.001	4.6 (3.4 – 6.2)	<0.001
Missing	34 (2.4%)	11 (3.3%)				
**Age (years)**						
18 – 29, n (%)	232 (16.7%)	90 (26.8%)	2.0 (1.4 – 2.7)	<0.001	1.6 (1.1 – 2.4)	0.019
30 – 39, n (%)	511 (36.7%)	100 (29.8%)	1		1	
40 – 49, n (%)	410 (29.5%)	86 (25.6%)	1.1 (0.8 - 1.5)	0.667	1.0 (0.7 – 1.4)	0.989
50 – 80, n (%)	185 (13.3%)	39 (11.6%)	1.1 (0.7 – 1.6)	0.720	1.1 (0.7 – 1.8)	0.575
Missing	53 (3.8%)	21 (6.3%)				
**Country of origin, n (%)**						
Germany	903 (64.9%)	202 (60.1%)	1		1	
Outside Germany	287 (20.6%)	84 (25.0%)	1.3 (1.0 – 1.7)	0.066	1.2 (0.8 – 1.7)	0.323
Missing	201 (14.5%)	50 (14.9%)				
**Annual gross income, n (%)**						
<30,000 €	314 (22.6%)	91 (27.1%)	1.4 (1.0 – 2.0)	0.039	1.0 (0.7 – 1.5)	0.971
30,000 – 49,000 €	424 (30.5%)	87 (25.9%)	1		1	
≥50,000 €	531 (38.2%)	121 (36.0%)	1.1 (0.8 – 1.5)	0.499	1.1 (0.8 – 1.6)	0.447
Missing	122 (8.8%)	37 (11.0%)				
**Gender, n (%)**						
Cisgender male	1,328 (95.5%)	310 (92.3%)	1		1	
Gender diverse	9 (0.6%)	5 (1.5%)	2.4 (0.8 – 7.2)	0.122	0.8 (0.2 – 3.4)	0.771
Missing	54 (3.9%)	21 (6.3%)				
**Test before starting PrEP, n(%)**						
No	9 (0.6%)	58 (17.3%)	1		[4]	
Yes	1,381 (99.3%)	270 (80.4%)	33.0 (16.1 – 67.3)	<0.001		
Missing	1 (0.1%)	8 (2.4%)				
**Payment for testing, n(%)**						
Cost coverage	755 (54.3%)	59 (17.6%)	1		[4]	
Self-payment	556 (40.0%)	60 (17.9%)	1.4 (0.9 – 2.0)	0.092		
Missing	80 (5.8%)	217 (64.6%)				
**Location of testing, n(%)**						
Physician	999 (71.8%)	86 (25.6%)	1		[4]	
Physician + other locations	167 (12.0%)	16 (4.8%)	1.1 (0.6 – 1.9)	0.707		
Only using checkpoints, self-tests, or other locations	197 (14.2%)	28 (8.3%)	1.7 (1.0 – 2.6)	0.030		
Missing	28 (2.0%)	206 (61.3%)				
**Number of anal/vaginal sex partners within the last 6 months, n (%)**						
0 – 3	169 (12.1%)	62 (18.5%)	2.1 (1.5 – 2.9)	<0.001	[4]	
4 – 10	396 (28.5%)	118 (35.1%)	1.7 (1.3 – 2.2)	<0.001		
> 10	770 (55.4%)	137 (40.8%)	1			
Missing	56 (4.0%)	19 (5.7%)				
**Condom use while taking PrEP, n(%)**						
Always / Often	1,076 (77.4%)	250 (74.4%)	1.1 (0.8 – 1.5)	0.548	[4]	
In about half of the times/sometimes/never	267 (19.2%)	68 (20.2%)	1			
Missing	48 (3.5%)	18 (5.4%)				

[1] Univariable logistic regression model; [2] Multivariable logistic regression model to investigate the association of informal PrEP use and infrequent testing behaviour including 1,135 participants with adequate and 276 participants with inadequate STI test frequency, adjusting for age, gender, country of origin, annual gross income, and type of PrEP use. [3] Wald test [4] Not included in the multivariable regression model (see appendix S1). CI: Confidence Interval, OR: Odds Ratio, PrEP: Pre-exposure prophylaxis, STI: sexually transmitted infection

Wave 2 (n = 2,069)

	Adequate STI test frequency	Inadequate STI test frequency	Univariable Analysis[1]		Multivariable Analysis[2]	
			OR (95% CI)	p-value[3]	OR (95% CI)	p-value[3]
Total (n)	1611	458				
**Source of current PrEP, n(%)**						
Medical Prescription	1,459 (90.6%)	313 (68.3%)	1		1	
Informal	143 (8.9%)	144 (31.4%)	4.7 (3.6 – 6.1)	<0.001	3.1 (2.2 – 4.3)	<0.001
Missing	9 (0.6%)	1 (0.2%)				
**Type of current PrEP use, n(%)**						
Daily	1,236 (76.7%)	194 (42.4%)	1		1	
On demand / intermittent	373 (23.2%)	262 (57.2%)	4.5 (3.6 – 5.6)	<0.001	3.9 (3.0 – 5.0)	<0.001
Missing	2 (0.1%)	2 (0.4%)				
**Age (years)**						
18 – 29, n (%)	354 (22.0%)	104 (22.7%)	1.1 (0.8 – 1.5)	0.495	1.2 (0.9 – 1.7)	0.233
30 – 39, n (%)	604 (37.5%)	161 (35.2%)	1		1	
40 – 49, n (%)	440 (27.3%)	123 (26.9%)	1.0 (0.8 – 1.4)	0.725	1.0 (0.7 – 1.3)	0.857
50 – 80, n (%)	213 (13.2%)	70 (15.3%)	1.2 (0.9 – 1.7)	0.201	1.0 (0.6 – 1.5)	0.884
Missing	- -	- -				
**Country of origin, n (%)**						
Germany	1,058 (65.7%)	278 (60.7%)	1		1	
Outside Germany	317 (19.7%)	108 (23.6%)	1.3 (1.0 – 1.7)	0.046	1.1 (0.8 – 1.4)	0.719
Missing	236 (14.6%)	72 (15.7%)				
**Annual gross income, n (%)**						
<30,000 €	443 (27.5%)	130 (28.4%)	1.2 (0.9 – 1.5)	0.336	1.0 (0.7 – 1.4)	0.940
30,000 – 49,000 €	439 (27.3%)	112 (24.5%)	1		1	
≥50,000 €	586 (36.4%)	163 (35.6%)	1.1 (0.8 – 1.4)	0.531	1.1 (0.8 – 1.5)	0.646
Missing	143 (8.9%)	53 (11.6%)				
**Gender, n (%)**						
Cisgender male	1,578 (98.0%)	454 (99.1%)	1		1	
Gender diverse	32 (2.0%)	4 (0.9%)	0.4 (0.2 – 1.2)	0.118	0.3 (0.1 – 0.9)	0.039
Missing	1 (0.1%)	0 (0.0%)				
**Test before starting PrEP, n(%)**						
No	14 (0.9%)	69 (15.1%)	1		[4]	
Yes	1,594 (98.9%)	382 (83.4%)	20.6 (11.5 – 36.9)	<0.001		
Missing	3 (0.2%)	7 (1.5%)				
**Payment for testing, n(%)**						
Cost coverage	908 (56.4%)	126 (27.5%)	1		[4]	
Self-payment	594 (36.9%)	114 (24.9%)	1.4 (1.1 – 1.8)	0.020		
Missing	109 (6.8%)	218 (47.6%)				
**Location of testing, n(%)**						
Physician	1,209 (75.0%)	189 (41.3%)	1		[4]	
Physician + other locations	168 (10.4%)	26 (5.7%)	1.0 (0.6 – 1.5)	0.964		
Only using checkpoints, self-tests, or other locations	184 (11.4%)	38 (8.3%)	1.3 (0.9 – 1.9)	0.152		
Missing	50 (3.1%)	205 (44.8%)				
**Number of anal/vaginal sex partners within the last 6 months, n (%)**						
0 – 3	198 (12.3%)	92 (20.1%)	2.0 (1.5 – 2.7)	<0.001	[4]	
4 – 10	552 (34.3%)	170 (37.1%)	1.3 (1.1 – 1.7)	0.015		
> 10	821 (51.0%)	189 (41.3%)	1			
Missing	40 (2.5%)	7 (1.5%)				
**Condom use while taking PrEP, n(%)**						
Always / Often	1,279 (79.4%)	318 (69.4%)	1.7 (1.3 – 2.2)	<0.001	[4]	
In about half of the times/sometimes/never	316 (19.6%)	134 (29.3%)	1			
Missing	16 (1.0%)	6 (1.3%)				

[1] Univariable logistic regression model [2] Multivariable logistic regression model to investigate the association of informal PrEP use and infrequent testing behaviour including 1,317 participants with adequate and 363 participants with inadequate STI test frequency, adjusting for age, gender, country of origin, annual gross income, and type of PrEP use. [3] Wald test [4] Not included in the multivariable regression model (see appendix S1). CI: Confidence Interval, OR: Odds Ratio, PrEP: Pre-exposure prophylaxis, STI: sexually transmitted infection

Appendix S6: Factors associated with renal function testing behaviour less frequent than recommended by guidelines among current PrEP users in Germany, 2018-2019; stratified by study waves

Wave 1 (n = 1,652)

	Adequate renal test frequency	Inadequate renal test frequency	Univariable Analysis[1]		Multivariable Analysis[2]	
			OR (95% CI)	p-value[3]	OR (95% CI)	p-value[3]
Total (n)	1177	475				
**Source of current PrEP, n(%)**						
Medical Prescription	1,034 (87.9%)	311 (65.5%)	1		1	
Informal	141 (12.0%)	163 (34.3%)	3.8 (3.0 – 5.0)	<0.001	2.4 (1.7 – 3.3)	<0.001
Missing	2 (0.2%)	1 (0.2%)				
**Type of current PrEP use, n(%)**						
Daily	987 (83.9%)	209 (44.0%)	1		1	
On demand / intermittent	187 (15.9%)	265 (55.8%)	6.7 (5.3 – 8.5)	<0.001	5.1 (3.9 – 6.8)	<0.001
Missing	3 (0.3%)	1 (0.2%)				
**Age (years)**						
18 – 29, n (%)	191 (16.2%)	125 (26.3%)	1.9 (1.4 – 2.5)	<0.001	1.6 (1.1 – 2.3)	0.012
30 – 39, n (%)	441 (37.5%)	156 (32.8%)	1		1	
40 – 49, n (%)	367 (31.2%)	123 (25.9%)	0.9 (0.7 – 1.2)	0.699	0.9 (0.6 – 1.2)	0.387
50 – 80, n (%)	164 (13.9%)	56 (11.8%)	1.0 (0.7 – 1.4)	0.845	0.9 (0.6 – 1.4)	0.662
Missing	14 (1.2%)	15 (3.2%)				
**Country of origin, n (%)**						
Germany	802 (68.1%)	287 (60.4%)	1		1	
Outside Germany	238 (20.2%)	120 (25.3%)	1.4 (1.1 – 1.8)	0.009	1.3 (0.9 – 1.7)	0.114
Missing	137 (11.6%)	68 (14.3%)				
**Annual gross income, n (%)**						
<30,000 €	263 (22.3%)	127 (26.7%)	1.3 (1.0 – 1.7)	0.079	0.8 (0.6 – 1.2)	0.337
30,000 – 49,000 €	375 (31.9%)	140 (29.5%)	1		1	
≥50,000 €	465 (39.5%)	168 (35.4%)	1.0 (0.7 – 1.3)	0.806	0.9 (0.7 – 1.3)	0.630
Missing	74 (6.3%)	40 (8.4%)				
**Gender, n (%)**						
Cisgender male	1,157 (98.3%)	452 (95.2%)	1		1	
Gender diverse	5 (0.4%)	8 (1.7%)	4.1 (1.3 – 12.6)	0.014	1.9 (0.5 – 7.9)	0.366
Missing	15 (1.3%)	15 (3.2%)				
**Test before starting PrEP, n(%)**						
No	4 (0.3%)	57 (12.0%)	40.7 (14.7 – 113.0)		[4]	
Yes	1,172 (99.6%)	410 (86.3%)	1			
Missing	1 (0.1%)	8 (1.7%)				
**Payment for testing, n(%)**						
Cost coverage	646 (54.9%)	142 (29.9%)	1		[4]	
Self-payment	486 (41.3%)	121 (25.5%)	1.1 (0.9 – 1.5)	0.365		
Missing	45 (3.8%)	212 (44.6%)				
**Location of testing, n(%)**						
Physician	894 (76.0%)	167 (35.2%)	1		[4]	
Physician + other locations	142 (12.1%)	39 (8.2%)	1.5 (1.0 – 2.2)	0.053		
Only using checkpoints, self-tests, or other locations	139 (11.8%)	75 (15.8%)	2.9 (2.1 – 4.0)	<0.001		
Missing	2 (0.2%)	194 (40.8%)				
**Number of anal/vaginal sex partners within the last 6 months, n (%)**						
0 – 3	139 (11.8%)	86 (18.1%)	2.0 (1.5 – 2.8)	<0.001	[4]	
4 – 10	337 (28.6%)	169 (35.6%)	1.6 (1.3 – 2.1)	<0.001		
> 10	682 (57.9%)	208 (43.8%)	1			
Missing	19 (1.6%)	12 (2.5%)				
**Condom use while taking PrEP, n(%)**						
Always / Often	944 (80.2%)	362 (76.2%)	1.2 (0.9 – 1.6)	0.135	[4]	
In about half of the times/sometimes/never	222 (18.9%)	104 (21.9%)	1			
Missing	11 (0.9%)	9 (1.9%)				

[1] Univariable logistic regression model; [2] Multivariable logistic regression model to investigate the association of informal PrEP use and infrequent testing behaviour including 989 participants with adequate and 391 participants with inadequate renal function test frequency, adjusting for age, gender, country of origin, annual gross income, and type of PrEP use. [3] Wald test [4] Not included in the multivariable regression model (see appendix S1). CI: Confidence Interval, OR: Odds Ratio, PrEP: Pre-exposure prophylaxis

Wave 2 (n = 1,966)

	Adequate renal test frequency	Inadequate renal test frequency	Univariable Analysis[1]		Multivariable Analysis[2]	
			OR (95% CI)	p-value[3]	OR (95% CI)	p-value[3]
Total (n)	1384	582				
**Source of current PrEP, n(%)**						
Medical Prescription	1,282 (92.6%)	403 (69.2%)	1		1	
Informal	94 (6.8%)	177 (30.4%)	6.0 (4.6 – 7.9)	<0.001	3.7 (2.6 – 5.1)	<0.001
Missing	8 (0.6%)	2 (0.3%)				
**Type of current PrEP use, n(%)**						
Daily	1,116 (80.6%)	249 (42.8%)	1		1	
On demand / intermittent	265 (19.1%)	332 (57.0%)	5.6 (4.5 – 6.9)	<0.001	4.7 (3.7 – 6.0)	<0.001
Missing	3 (0.2%)	1 (0.2%)				
**Age (years)**						
18 – 29, n (%)	284 (20.5%)	141 (24.2%)	1.2 (0.9 – 1.5)	0.286	1.2 (0.9 – 1.7)	0.251
30 – 39, n (%)	505 (36.5%)	218 (37.5%)	1		1	
40 – 49, n (%)	400 (28.9%)	137 (23.5%)	0.8 (0.6 – 1.0)	0.070	0.7 (0.5 – 1.0)	0.050
50 – 80, n (%)	195 (14.1%)	86 (14.8%)	1.0 (0.8 – 1.4)	0.889	0.9 (0.6 – 1.3)	0.414
Missing	- -	- -				
**Country of origin, n (%)**						
Germany	972 (70.2%)	340 (58.4%)	1		1	
Outside Germany	249 (18.0%)	146 (25.1%)	1.7 (1.3 – 2.1)	<0.001	1.4 (1.1 – 1.8)	0.020
Missing	163 (11.8%)	96 (16.5%)				
**Annual gross income, n (%)**						
<30,000 €	370 (26.7%)	175 (30.1%)	1.2 (0.9 – 1.6)	0.159	1.1 (0.8 – 1.5)	0.629
30,000 – 49,000 €	385 (27.8%)	151 (25.9%)	1		1	
≥50,000 €	532 (38.4%)	210 (36.1%)	1.0 (0.8 – 1.3)	0.959	1.0 (0.8 – 1.4)	0.843
Missing	97 (7.0%)	46 (7.9%)				
**Gender, n (%)**						
Cisgender male	1,359 (98.2%)	573 (98.5%)	1		1	
Gender diverse	24 (1.7%)	9 (1.5%)	0.9 (0.4 – 1.9)	0.766	0.6 (0.3 – 1.7)	0.367
Missing	1 (0.1%)	0 (0.0%)				
**Test before starting PrEP, n(%)**						
No	10 (0.7%)	70 (12.0%)	19.1 (9.8 – 37.3)	<0.001	[4]	
Yes	1,373 (99.2%)	504 (86.6%)	1			
Missing	1 (0.1%)	8 (1.4%)				
**Payment for testing, n(%)**						
Cost coverage	784 (56.6%)	209 (35.9%)	1		[4]	
Self-payment	542 (39.2%)	151 (25.9%)	1.0 (0.8 – 1.3)	0.715		
Missing	58 (4.2%)	222 (38.1%)				
**Location of testing, n(%)**						
Physician	1,108 (80.1%)	246 (42.3%)	1		[4]	
Physician + other locations	140 (10.1%)	47 (8.1%)	1.5 (1.1 – 2.2)	0.024		
Only using checkpoints, self-tests, or other locations	128 (9.2%)	86 (14.8%)	3.0 (2.2 – 4.1)	<0.001		
Missing	8 (0.6%)	203 (34.9%)				
**Number of anal/vaginal sex partners within the last 6 months, n (%)**						
0 – 3	163 (11.8%)	118 (20.3%)	2.4 (1.8 – 3.2)	<0.001	[4]	
4 – 10	459 (33.2%)	230 (39.5%)	1.6 (1.3 – 2.0)	<0.001		
> 10	730 (52.7%)	222 (38.1%)	1			
Missing	32 (2.3%)	12 (2.1%)				
**Condom use while taking PrEP, n(%)**						
Always / Often	1,105 (79.8%)	417 (71.6%)	1.6 (1.3 – 2.0)	<0.001	[4]	
In about half of the times/sometimes/never	263 (19.0%)	161 (27.7%)	1			
Missing	16 (1.2%)	4 (0.7%)				

[1] Univariable logistic regression model [2] Multivariable logistic regression model to investigate the association of informal PrEP use and infrequent testing behaviour including 1,166 participants with adequate and 467 participants with inadequate renal function test frequency, adjusting for age, gender, country of origin, annual gross income, and type of PrEP use. [3] Wald test [4] Not included in the multivariable regression model (see appendix S1). CI: Confidence Interval, OR: Odds Ratio, PrEP: Pre-exposure prophylaxis,

Appendix S7: Comparison of study participants with and without missing data among current PrEP users in Germany, 2018-2019

Regression model for insufficient HIV testing frequency (n = 3,892)

	Included in regression model on insufficient HIV testing frequency		Excluded in regression model on insufficient HIV testing frequency due to missing data	
Total (n)	3146		746	
**HIV testing frequency**				
Sufficient according to guidelines	2,338	(74.3%)	531	(71.2%)
Not sufficient according to guidelines	808	(25.7%)	215	(28.8%)
**Test before starting PrEP, n(%)**				
No	107	(3.4%)	44	(5.9%)
Yes	3,023	(96.6%)	698	(94.1%)
Missing	16	-	4	-
**Source of current PrEP, n(%)**				
Medical Prescription	2,657	(84.5%)	598	(81.6%)
Informal	489	(15.5%)	135	(18.4%)
Missing	0	-	13	-
**Type of current PrEP use, n(%)**				
Daily	2,243	(71.3%)	456	(66.8%)
On demand / intermittent	903	(28.7%)	227	(33.2%)
Missing	0	-	63	-
**Age (years)**				
18 – 29, n (%)	661	(21.0%)	134	(20.4%)
30 – 39, n (%)	1,169	(37.2%)	231	(35.2%)
40 – 49, n (%)	904	(28.7%)	177	(26.9%)
50 – 80, n (%)	412	(13.1%)	115	(17.5%)
Missing	0	-	89	-
**Country of origin, n (%)**				
Germany	2,379	(75.6%)	113	(75.3%)
Outside Germany	767	(24.4%)	37	(24.7%)
Missing	0	-	596	-
**Annual gross income, n (%)**				
<30,000 €	883	(28.1%)	109	(30.4%)
30,000 – 49,000 €	974	(31.0%)	117	(32.6%)
≥50,000 €	1,289	(41.0%)	133	(37.0%)
Missing	0	-	387	-
**Gender, n (%)**				
Cisgender male	3,105	(98.7%)	643	(98.2%)
Gender diverse	41	(1.3%)	12	(1.8%)
Missing	0	-	91	-
**Payment for testing, n(%)**				
Cost coverage	1,573	(57.7%)	307	(60.0%)
Self-payment	1,151	(42.3%)	205	(40.0%)
Missing	422	-	234	-
**Location of testing, n(%)**				
Physician	2,136	(75.4%)	409	(74.8%)
Physician + other locations	326	(11.5%)	58	(10.6%)
Only using checkpoints, self-tests, or other locations	370	(13.1%)	80	(14.6%)
Missing	314	-	199	-
**Number of anal/vaginal sex partners within the last 6 months, n (%)**				
0 – 3	446	(14.4%)	98	(15.0%)
4 – 10	1,047	(33.8%)	216	(33.0%)
> 10	1,606	(51.8%)	340	(52.0%)
Missing	47	-	92	-
**Condom use while taking PrEP, n(%)**				
Always / Often	2,475	(79.3%)	506	(76.1%)
In about half of the times/sometimes/never	648	(20.7%)	159	(23.9%)
Missing	23	-	81	-

HIV: human immunodeficiency virus, PrEP: Pre-exposure prophylaxis,

Regression model for insufficient STI testing frequency (n = 3,796)

	Included in regression model on insufficient STI testing frequency	Excluded in regression model on insufficient STI testing frequency due to missing data
Total (n)	3091	705
**STI testing frequency**		
Sufficient according to guidelines	2,452 (79.3%)	550 (78.0%)
Not sufficient according to guidelines	639 (20.7%)	155 (22.0%)
**Test before starting PrEP, n(%)**		
No	109 (3.5%)	41 (5.8%)
Yes	2,967 (96.5%)	660 (94.2%)
Missing	15 -	4 -
**Source of current PrEP, n(%)**		
Medical Prescription	2,603 (84.2%)	568 (82.1%)
Informal	488 (15.8%)	124 (17.9%)
Missing	0 -	13 -
**Type of current PrEP use, n(%)**		
Daily	2,199 (71.1%)	440 (67.1%)
On demand / intermittent	892 (28.9%)	216 (32.9%)
Missing	0 -	49 -
**Age (years)**		
18 – 29, n (%)	653 (21.1%)	127 (20.1%)
30 – 39, n (%)	1,151 (37.2%)	225 (35.7%)
40 – 49, n (%)	889 (28.8%)	170 (26.9%)
50 – 80, n (%)	398 (12.9%)	109 (17.3%)
Missing	0 -	74 -
**Country of origin, n (%)**		
Germany	2,333 (75.5%)	108 (74.0%)
Outside Germany	758 (24.5%)	38 (26.0%)
Missing	0 -	559 -
**Annual gross income, n (%)**		
<30,000 €	873 (28.2%)	105 (30.0%)
30,000 – 49,000 €	950 (30.7%)	112 (32.0%)
≥50,000 €	1,268 (41.0%)	133 (38.0%)
Missing	0 -	355 -
**Gender, n (%)**		
Cisgender male	3,051 (98.7%)	619 (98.4%)
Gender diverse	40 (1.3%)	10 (1.6%)
Missing	0 -	76 -
**Payment for testing, n(%)**		
Cost coverage	1,548 (57.9%)	300 (60.4%)
Self-payment	1,127 (42.1%)	197 (39.6%)
Missing	416 -	208 -
**Location of testing, n(%)**		
Physician	2,088 (75.2%)	395 (74.4%)
Physician + other locations	320 (11.5%)	57 (10.7%)
Only using checkpoints, self-tests, or other locations	368 (13.3%)	79 (14.9%)
Missing	315 -	174 -
**Number of anal/vaginal sex partners within the last 6 months, n (%)**		
0 – 3	432 (14.2%)	89 (14.2%)
4 – 10	1,025 (33.7%)	211 (33.6%)
> 10	1,589 (52.2%)	328 (52.2%)
Missing	45 -	77 -
**Condom use while taking PrEP, n(%)**		
Always / Often	2,434 (79.3%)	489 (76.5%)
In about half of the times/sometimes/never	635 (20.7%)	150 (23.5%)
Missing	22 -	66 -

STI: sexually transmitted infection, PrEP: Pre-exposure prophylaxis,

Regression model for insufficient renal function testing frequency (n = 3,618)

	Included in regression model on insufficient renal testing frequency	Excluded in regression model on insufficient renal testing frequency due to missing data
Total (n)	3013	605
**Renal function testing frequency**		
Sufficient according to guidelines	2,155 (71.5%)	406 (67.1%)
Not sufficient according to guidelines	858 (28.5%)	199 (32.9%)
**Test before starting PrEP, n(%)**		
No	105 (3.5%)	36 (6.0%)
Yes	2,892 (96.5%)	567 (94.0%)
Missing	16 -	2 -
**Source of current PrEP, n(%)**		
Medical Prescription	2,540 (84.3%)	490 (82.8%)
Informal	473 (15.7%)	102 (17.2%)
Missing	0 -	13 -
**Type of current PrEP use, n(%)**		
Daily	2,159 (71.7%)	402 (67.3%)
On demand / intermittent	854 (28.3%)	195 (32.7%)
Missing	0 -	8 -
**Age (years)**		
18 – 29, n (%)	628 (20.8%)	113 (19.6%)
30 – 39, n (%)	1,117 (37.1%)	203 (35.2%)
40 – 49, n (%)	870 (28.9%)	157 (27.3%)
50 – 80, n (%)	398 (13.2%)	103 (17.9%)
Missing	0 -	29 -
**Country of origin, n (%)**		
Germany	2,295 (76.2%)	106 (75.2%)
Outside Germany	718 (23.8%)	35 (24.8%)
Missing	0 -	464 -
**Annual gross income, n (%)**		
<30,000 €	828 (27.5%)	107 (30.7%)
30,000 – 49,000 €	939 (31.2%)	112 (32.2%)
≥50,000 €	1,246 (41.4%)	129 (37.1%)
Missing	0 -	257 -
**Gender, n (%)**		
Cisgender male	2,977 (98.8%)	564 (98.3%)
Gender diverse	36 (1.2%)	10 (1.7%)
Missing	0 -	31 -
**Payment for testing, n(%)**		
Cost coverage	1,493 (57.4%)	288 (60.0%)
Self-payment	1,108 (42.6%)	192 (40.0%)
Missing	412 -	125 -
**Location of testing, n(%)**		
Physician	2,036 (75.4%)	379 (74.2%)
Physician + other locations	313 (11.6%)	55 (10.8%)
Only using checkpoints, self-tests, or other locations	351 (13.0%)	77 (15.1%)
Missing	313 -	94 -
**Number of anal/vaginal sex partners within the last 6 months, n (%)**		
0 – 3	420 (14.2%)	86 (14.9%)
4 – 10	1,003 (33.8%)	192 (33.3%)
> 10	1,544 (52.0%)	298 (51.7%)
Missing	46 -	29 -
**Condom use while taking PrEP, n(%)**		
Always / Often	2,380 (79.5%)	448 (76.5%)
In about half of the times/sometimes/never	612 (20.5%)	138 (23.5%)
Missing	21 -	19 -

PrEP: Pre-exposure prophylaxis,

Appendix S8: Factors associated with HIV testing behaviour less frequent than recommended by guidelines among current PrEP users in Germany, 2018-2019; excluding participants receiving PrEP through a clinical trial (n = 3,767)

	Adequate HIV test frequency	Inadequate HIV test frequency	Univariable Analysis[1]		Multivariable Analysis[2]	
			OR (95% CI)	p-value[3]	OR (95% CI)	p-value[3]
Total (n)	2,750	1,017				
**Source of current PrEP, n(%)**						
Medical Prescription	2,474 (90.0%)	656 (64.5%)	1		1	
Informal	265 (9.6%)	359 (35.3%)	5.1 (4.3 – 6.1)	<0.001	3.5 (2.8 – 4.4)	<0.001
Missing	11 (0.4%)	2 (0.2%)	-			
**Type of current PrEP use, n(%)**						
Daily	2,212 (80.4%)	380 (37.4%)	1		1	
On demand / intermittent	508 (18.5%)	612 (60.2%)	7.0 (6.0 – 8.2)	<0.001	5.7 (4.8 – 6.9)	<0.001
Missing	30 (1.1%)	25 (2.5%)	-			
**Age (years)**						
18 – 29, n (%)	541 (19.7%)	238 (23.4%)	1.3 (1.1 – 1.6)	0.009	1.2 (0.9 – 1.5)	0.157
30 – 39, n (%)	1,003 (36.5%)	340 (33.4%)	1		1	
40 – 49, n (%)	793 (28.8%)	254 (25.0%)	0.9 (0.8 – 1.1)	0.553	0.8 (0.6 – 1.0)	0.048
50 – 80, n (%)	367 (13.3%)	149 (14.7%)	1.2 (1.0 – 1.5)	0.119	1.0 (0.8 – 1.4)	0.913
Missing	46 (1.7%)	36 (3.5%)	-			
**Country of origin, n (%)**						
Germany	1,824 (66.3%)	598 (58.8%)	1		1	
Outside Germany	527 (19.2%)	243 (23.9%)	1.4 (1.2 – 1.7)	<0.001	1.2 (1.0 – 1.5)	0.117
Missing	399 (14.5%)	176 (17.3%)	-			
**Annual gross income, n (%)**						
<30,000 €	677 (24.6%)	279 (27.4%)	1.3 (1.1 – 1.6)	0.007	1.1 (0.8 – 1.4)	0.572
30,000 – 49,000 €	799 (29.1%)	251 (24.7%)	1		1	
≥50,000 €	1,019 (37.1%)	371 (36.5%)	1.2 (1.0 – 1.4)	0.118	1.3 (1.0 – 1.6)	0.031
Missing	255 (9.3%)	116 (11.4%)	-			
**Gender, n (%)**						
Cisgender male	2,671 (97.1%)	961 (94.5%)	1		1	
Gender diverse	31 (1.1%)	20 (2.0%)	1.8 (1.0 – 3.2)	0.044	1.0 (0.5 – 2.0)	0.932
Missing	48 (1.7%)	36 (3.5%)	-			
**Test before starting PrEP, n(%)**						
Yes	2,725 (99.1%)	872 (85.7%)	1		[4]	
No	23 (0.8%)	127 (12.5%)	17.3 (11.0 – 27.1)	<0.001		
Missing	2 (0.1%)	18 (1.8%)	-			
**Payment for testing, n(%)**						
Cost coverage	1,455 (52.9%)	320 (31.5%)	1		[4]	
Self-payment	1,113 (40.5%)	239 (23.5%)	1.0 (0.8 – 1.2)	0.800		
Missing	182 (6.6%)	458 (45.0%)	-			
**Location of testing, n(%)**						
Physician	2,047 (74.4%)	391 (38.4%)	1		[4]	
Physician + other locations	319 (11.6%)	62 (6.1%)	1.0 (0.8 – 1.4)	0.907		
Only using checkpoints, self-tests, or other locations	301 (10.9%)	141 (13.9%)	2.5 (2.0 – 3.1)	<0.001		
Missing	83 (3.0%)	423 (41.6%)	-			
**Number of anal/vaginal sex partners within the last 6 months, n (%)**						
0 – 3	314 (11.4%)	214 (21.0%)	2.6 (2.1 – 3.2)	<0.001	[4]	
4 – 10	869 (31.6%)	371 (36.5%)	1.6 (1.4 – 1.9)	<0.001		
> 10	1,483 (53.9%)	384 (37.8%)	1			
Missing	84 (3.1%)	48 (4.7%)	-			
**Condom use while taking PrEP, n(%)**						
Always / Often	541 (19.7%)	254 (25.0%)	1.4 (1.2 – 1.6)	<0.001	[4]	
In about half of the times/sometimes/never	2,148 (78.1%)	727 (71.5%)	1			
Missing	61 (2.2%)	36 (3.5%)	-			

[1] Univariable logistic regression model. [2] Multivariable logistic regression model to investigate the association of informal PrEP use and infrequent testing behaviour including 2,243 participants with adequate and 806 participants with inadequate HIV test frequency, adjusting for age, gender, country of origin, annual gross income, and type of PrEP use. [3] Wald test [4] Not included in the multivariable regression model (see appendix S1). CI: Confidence Interval, OR: Odds Ratio, PrEP: Pre-exposure prophylaxis, HIV: human immunodeficiency virus

Appendix S9: Factors associated with STI testing behaviour less frequent than recommended by guidelines among current PrEP users in Germany, 2018-2019; excluding participants receiving PrEP through a clinical trial (n = 3672)

	Adequate STI test frequency	Inadequate STI test frequency	Univariable Analysis[1]		Multivariable Analysis[2]	
			OR (95% CI)	p-value[3]	OR (95% CI)	p-value[3]
Total (n)	2,883	789				
**Source of current PrEP, n(%)**						
Medical Prescription	2,531 (87.8%)	516 (65.4%)	1		1	
Informal	340 (11.8%)	272 (34.5%)	3.9 (3.3 – 4.7)	<0.001	2.6 (2.1 – 3.3)	<0.001
Missing	12 (0.4%)	1 (0.1%)				
**Type of current PrEP use, n(%)**						
Daily	2,210 (76.7%)	322 (40.8%)	1		1	
On demand / intermittent	642 (22.3%)	456 (57.8%)	4.9 (4.1 – 5.8)	<0.001	4.1 (3.4 – 4.9)	<0.001
Missing	31 (1.1%)	11 (1.4%)				
**Age (years)**						
18 – 29, n (%)	570 (19.8%)	194 (24.6%)	1.4 (1.1 – 1.7)	0.002	1.4 (1.1 – 1.8)	0.017
30 – 39, n (%)	1,061 (36.8%)	258 (32.7%)	1		1	
40 – 49, n (%)	817 (28.3%)	208 (26.4%)	1.0 (0.9 – 1.3)	0.659	1.0 (0.8 – 1.2)	0.829
50 – 80, n (%)	387 (13.4%)	109 (13.8%)	1.2 (0.9 – 1.5)	0.254	1.0 (0.8 – 1.4)	0.767
Missing	48 (1.7%)	20 (2.5%)				
**Country of origin, n (%)**						
Germany	1,891 (65.6%)	480 (60.8%)	1		1	
Outside Germany	572 (19.8%)	190 (24.1%)	1.3 (1.1 – 1.6)	0.006	1.1 (0.9 – 1.4)	0.323
Missing	420 (14.6%)	119 (15.1%)				
**Annual gross income, n (%)**						
<30,000 €	723 (25.1%)	219 (27.8%)	1.3 (1.0 – 1.6)	0.042	1.0 (0.8 – 1.3)	0.963
30,000 – 49,000 €	822 (28.5%)	199 (25.2%)	1		1	
≥50,000 €	1,086 (37.7%)	283 (35.9%)	1.1 (0.9 – 1.3)	0.477	1.1 (0.9 – 1.4)	0.455
Missing	252 (8.7%)	88 (11.2%)				
**Gender, n (%)**						
Cisgender male	2,794 (96.9%)	760 (96.3%)	1		1	
Gender diverse	39 (1.4%)	9 (1.1%)	0.8 (0.4 – 1.8)	0.659	0.4 (0.2 – 1.0)	0.060
Missing	50 (1.7%)	20 (2.5%)				
**Test before starting PrEP, n(%)**						
Yes	2,856 (99.1%)	648 (82.1%)	1		[4]	
No	23 (0.8%)	126 (16.0%)	24.1 (15.4 – 38.0)	<0.001		
Missing	4 (0.1%)	15 (1.9%)				
**Payment for testing, n(%)**						
Cost coverage	1,560 (54.1%)	183 (23.2%)	1		[4]	
Self-payment	1,147 (39.8%)	173 (21.9%)	1.3 (1.0 – 1.6)	0.026		
Missing	176 (6.1%)	433 (54.9%)				
**Location of testing, n(%)**						
Physician	2,104 (73.0%)	272 (34.5%)	1		[4]	
Physician + other locations	332 (11.5%)	42 (5.3%)	1.0 (0.7 – 1.4)	0.902		
Only using checkpoints, self-tests, or other locations	373 (12.9%)	66 (8.4%)	1.4 (1.0 – 1.8)	0.034		
Missing	74 (2.6%)	409 (51.8%)				
**Number of anal/vaginal sex partners within the last 6 months, n (%)**						
0 – 3	352 (12.2%)	153 (19.4%)	2.0 (1.6 – 2.5)	<0.001	[4]	
4 – 10	926 (32.1%)	287 (36.4%)	1.4 (1.2 – 1.7)	<0.001		
> 10	1,514 (52.5%)	324 (41.1%)	1			
Missing	91 (3.2%)	25 (3.2%)				
**Condom use while taking PrEP, n(%)**						
Always / Often	573 (19.9%)	200 (25.3%)	1.4 (1.2 – 1.7)	0.001	[4]	
In about half of the times/sometimes/never	2,251 (78.1%)	566 (71.7%)	1			
Missing	59 (2.0%)	23 (2.9%)				

[1] Univariable logistic regression model. [2] Multivariable logistic regression model to investigate the association of informal PrEP use and infrequent testing behaviour including 2,356 participants with adequate and 638 participants with inadequate STI test frequency, adjusting for age, gender, country of origin, annual gross income, and type of PrEP use. [3] Wald test [4] Not included in the multivariable regression model (see appendix S1). CI: Confidence Interval, OR: Odds Ratio, PrEP: Pre-exposure prophylaxis, STI: sexually transmitted infection

Appendix S10: Factors associated with renal function testing behaviour less frequent than recommended by guidelines among current PrEP users in Germany, 2018-2019; excluding participants receiving PrEP through a clinical trial (n = 3,502)

	Adequate renal test frequency	Inadequate renal test frequency	Univariable Analysis[1]		Multivariable Analysis[2]	
			OR (95% CI)	p-value[3]	OR (95% CI)	p-value[3]
Total (n)	2,454	1,048				
**Source of current PrEP, n(%)**						
Medical Prescription	2,209 (90.0%)	705 (67.3%)	1		1	
Informal	235 (9.6%)	340 (32.4%)	4.5 (3.8 – 5.5)	<0.001	2.9 (2.3 – 3.6)	<0.001
Missing	10 (0.4%)	3 (0.3%)				
**Type of current PrEP use, n(%)**						
Daily	2,003 (81.6%)	452 (43.1%)	1		1	
On demand / intermittent	446 (18.2%)	594 (56.7%)	5.9 (5.0 – 6.9)	<0.001	4.7 (3.9 – 5.7)	<0.001
Missing	5 (0.2%)	2 (0.2%)				
**Age (years)**						
18 – 29, n (%)	461 (18.8%)	265 (25.3%)	1.4 (1.2 – 1.7)	0.001	1.4 (1.1 – 1.7)	0.012
30 – 39, n (%)	897 (36.6%)	367 (35.0%)	1		1	
40 – 49, n (%)	733 (29.9%)	260 (24.8%)	0.9 (0.7 – 1.0)	0.133	0.8 (0.6 – 1.0)	0.050
50 – 80, n (%)	349 (14.2%)	141 (13.5%)	1.0 (0.8 – 1.2)	0.914	0.9 (0.7 – 1.2)	0.381
Missing	14 (0.6%)	15 (1.4%)				
**Country of origin, n (%)**						
Germany	1,707 (69.6%)	624 (59.5%)	1		1	
Outside Germany	457 (18.6%)	264 (25.2%)	1.6 (1.3 – 1.9)	<0.001	1.4 (1.1 – 1.7)	0.004
Missing	290 (11.8%)	160 (15.3%)				
**Annual gross income, n (%)**						
<30,000 €	605 (24.7%)	296 (28.2%)	1.2 (1.0 – 1.5)	0.056	0.9 (0.7 – 1.2)	0.632
30,000 – 49,000 €	719 (29.3%)	291 (27.8%)	1		1	
≥50,000 €	967 (39.4%)	376 (35.9%)	1.0 (0.8 – 1.2)	0.664	1.0 (0.8 – 1.2)	0.653
Missing	163 (6.6%)	85 (8.1%)				
**Gender, n (%)**						
Cisgender male	2,411 (98.2%)	1,016 (96.9%)	1		1	
Gender diverse	27 (1.1%)	17 (1.6%)	1.5 (0.8 – 2.8)	0.198	1.0 (0.4 – 2.1)	0.926
Missing	16 (0.7%)	15 (1.4%)				
**Test before starting PrEP, n(%)**						
Yes	2,438 (99.3%)	906 (86.5%)	1		[4]	
No	14 (0.6%)	126 (12.0%)	24.2 (13.9 – 42.3)	<0.001		
Missing	2 (0.1%)	16 (1.5%)				
**Payment for testing, n(%)**						
Cost coverage	1,336 (54.4%)	343 (32.7%)	1		[4]	
Self-payment	1,024 (41.7%)	272 (26.0%)	1.0 (0.9 – 1.2)	0.709		
Missing	94 (3.8%)	433 (41.3%)				
**Location of testing, n(%)**						
Physician	1,905 (77.6%)	406 (38.7%)	1		[4]	
Physician + other locations	279 (11.4%)	86 (8.2%)	1.4 (1.1 – 1.9)	0.006		
Only using checkpoints, self-tests, or other locations	261 (10.6%)	160 (15.3%)	2.9 (2.3 – 3.6)	<0.001		
Missing	9 (0.4%)	396 (37.8%)				
**Number of anal/vaginal sex partners within the last 6 months, n (%)**						
0 – 3	291 (11.9%)	200 (19.1%)	2.2 (1.7 – 2.7)	<0.001	[4]	
4 – 10	776 (31.6%)	397 (37.9%)	1.6 (1.4 – 1.9)	<0.001		
> 10	1,336 (54.4%)	427 (40.7%)	1			
Missing	51 (2.1%)	24 (2.3%)				
**Condom use while taking PrEP, n(%)**						
Always / Often	476 (19.4%)	263 (25.1%)	1.4 (1.2 – 1.7)	<0.001	[4]	
In about half of the times/sometimes/never	1,951 (79.5%)	772 (73.7%)	1			
Missing	27 (1.1%)	13 (1.2%)				

[1] Univariable logistic regression model [2] Multivariable logistic regression model to investigate the association of informal PrEP use and infrequent testing behaviour including 2,065 participants with adequate and 853 participants with inadequate renal function test frequency, adjusting for age, gender, country of origin, annual gross income, and type of PrEP use. [3] Wald test [4] Not included in the multivariable regression model (see appendix S1). CI: Confidence Interval, OR: Odds Ratio, PrEP: Pre-exposure prophylaxis

Appendix S11: Survey questions included in this analysis

Questions for current PrEP users included in the analysis

Wave 1

Question	Answer options
**PreP usage**	
Are you currently taking or have you ever taken drugs for pre-exposure prophylaxis (PrEP) against HIV?	• Yes, I am taking PrEP on a daily basis.<br>• Yes, I take it intermittently when I think I need it<br>• Yes, I used to take PrEP, but I permanently stopped<br>• No

General information	
How old are you?	Drop-down (range 18 to >80)
What gender do you identify with?	• Male<br>• Female<br>• Trans*<br>• Intersexual<br>• Other
What is your approximate gross income per year?<br><br>*Gross income: income before deduction of taxes and social security contributions*	• Less than 30,000€<br>• 30,000 – 39,000€<br>• 40,000 – 49,000€<br>• 50,000 – 59,000€<br>• 60,000 – 69,000€<br>• 70,000€ or more<br>• I don't know
In which country were you born?	Drop-down
Where did you hear about this study?	Select all that apply<br>• Planetromeo<br>• Grindr<br>• Hornet<br>• Checkpoint<br>• through friends
With how many different male partners have you had anal sex within the last 6 months?	• 0<br>• 1<br>• 2-3<br>• 4-5<br>• 6-10<br>• more than 10<br>• I don't know

How happy are you with your sex life at the moment?	• Very happy<br>• Happy<br>• I'm not sure<br>• Unhappy<br>• Very unhappy<br>• Sex is not important for me at the moment

**Experiences with PrEP**	
How are you taking PrEP?	• I am taking PrEP permanently<br>• I am taking PrEP intermittently / occasionally (e.g. during pride season or on holidays)<br>• I am taking PrEP on demand when I have risky sexual encounters<br>• Other:______
When did you first start taking PrEP?	• Less than 3 months ago<br>• 3-6 months ago<br>• 7-12 months ago<br>• 13-24 months ago<br>• More than 24 months ago
Within the last 12 months, for how many months did you take PrEP?	Drop-down: 0-12 months
How often do you take PrEP on average per month<br><br>If you take PrEP intermittently / occasionally please refer to the use of PrEP in a month when you are taking PrEP	• On all or almost all days (26 days or more)<br>• On many or most days (12-25 days)<br>• On a few days (1-11 days)
Where did you obtain PrEP from?<br><br>(please indicate the last source where you obtained PrEP)	• German pharmacy – prescription for about 50€ (Blister prescription)<br>• German pharmacy prescription for more than 50€, but less than 100€<br>• German pharmacy – prescription for 500€ - 800€<br>• Friends<br>• Dealer<br>• Sex Party<br>• Research Study<br>• Internet / ordered online from another country<br>• I regularly fly to another country where I get PrEP<br>• I used PEP-medication as PrEP<br>• Other: _________

Since October 2017 PrEP in Germany is available at prices between 50€ and 70€ in pharmacies. Is the price affordable for you?	• Yes, I can afford PrEP<br>• Yes, but it is hard for me to come up with the money to buy PrEP<br>• No, I can't afford PrEP at that price<br>• I don't know

**Clinical testing**	
Have you gotten tested **before** starting PrEP medications (e.g. HIV test, STI tests, …)?	• Yes<br>• No<br>• I don't know
Which of the following tests were administered to you **before starting PrEP**?	Select all that apply:<br>• Test for HIV<br>• Hepatitis B<br>• Hepatitis C<br>• Syphilis<br>• Gonorrhea<br>• Chlamydia<br>• Mycoplasma<br>• Kidney function<br>• Other tests<br>Don't remember
Have you been tested for HIV, other sexually transmitted infections (STI), or have been checked for your kidney function **while taking PrEP**?	• Yes<br>• No<br>• Don't know
**How often** do you get tested for HIV **while you are on PrEP**?	• At least once every 3 months<br>• At least once every 6 months<br>• At least once per year<br>• Less than once every year<br>• I don't get tested for this<br>• I don't know
Why aren't you getting tested for HIV?<br><br>Only if "not at all" was selected for previous question.	Select all that apply<br>• I can't afford the test<br>• The test was not offered to me<br>• I don't have time to take the test<br>• I don't want to take the test<br>• I don't think I would benefit from this test<br>• I didn't know I was supposed to take this test<br>• Other reason:
**How often** do you get tested for STIs **while you are on PrEP**, e.g. gonorrhoea, syphilis, chlamydia?	• At least once every 3 months<br>• At least once every 6 months<br>• At least once per year<br>• Less than once every year<br>• I don't get tested for this

	• I don't know
Why aren't you getting tested for sexually transmitted infections (STI)?<br><br>Only if "not at all" was selected for previous question.	Select all that apply<br>• I can't afford the tests<br>• The tests were not offered to me<br>• I don't have time to take the tests<br>• I don't want to take the tests<br>• I don't think I would benefit from these tests<br>• I didn't know I was supposed to take this test<br>• Other reason:
For which of the following STIs have you tested positive? / Which STIs have you ever been diagnosed with?	Select all that apply:<br>• Syphilis<br>• Gonorrhea<br>• Chlamydia<br>• Genital warts / HPV<br>• Hepatitis A<br>• Hepatitis B<br>• Hepatitis C<br>• Mycoplasma<br>• Other: ______<br>• None<br>• I don't remember
Follow-up questions for every option in the previous question that was declared positive:<br>• When were you last diagnosed with a new syphilis infection?<br>• When were you last tested positive for gonorrhea?<br>• When were you last tested positive for chlamydia?<br>• When were you first diagnosed with genital warts/ HPV?<br>• When were you diagnosed with hepatitis A?<br>• When were you diagnosed with hepatitis B?<br>• When were you last diagnosed with hepatitis C? then: Did you ever have hepatitis C before (which either cleared spontaneously or was successfully treated)?<br>• When were you last tested positive for mycoplasma?<br>• When were you last tested positive for other: _____	• Within the last 7 days<br>• Within the last 4 weeks<br>• Within the last 6 months<br>• Within the last 12 months<br>• Within the last 24 months<br>• More than 24 months ago<br><br>Answers HCV-Followup Question<br>• No<br>• Yes, once<br>• Yes, more than once
**How often** do you get tested for your kidney function **while you are on PrEP**?	• At least once every 3 months<br>• At least once every 6 months<br>• At least once per year<br>• Less than once every year<br>• I am not getting tested for this<br>• I don't know
Why aren't you getting a test to check your kidney function?	Check all that apply<br>• I can't afford the test

Only if “not at all” was selected for previous question	• The test was not offered to me<br>• I don't have time to take the test<br>• I don't want to take the test<br>• I don't think I would benefit from this test<br>• - I didn’t know I was supposed to take this test<br>• other reason:
**Where** do you get tested while you are using PrEP (e.g. for HIV, STIs, or kidney function)?	Select all that apply:<br>• At the doctor who prescribes the medication<br>• At a community based testing site / anonymous testing clinic<br>• Other: _______
**How much** do you pay when you get these tests?	• I don’t pay anything at the doctor / it’s covered by my health insurance<br>• Less than 50€ for all tests<br>• About 50-100€ for all tests<br>• More than 100€ for all tests<br>• I don’t know

**Drivers for PrEP use**	
Why do you take PrEP?	Select all that apply:<br>• I don’t want to use condoms and still want to protect myself<br>• My partner doesn’t want to use condoms and I still want to protect myself<br>• Sex without a condom is expected by my peers and I still want to protect myself<br>• I want to protect myself against HIV in case the condom breaks<br>• Sometimes condoms aren’t available and I still want to be protected<br>• It’s more convenient since I don’t have to talk about or negotiate condom use<br>• I cannot get an erection when I use a condom but I still want to protect myself<br>• My partner is HIV positive and I want to protect myself

	• I use condoms and I want additional protection<br>• Other: __________

**Condom use and sharing PrEP use with others**	
How often do you use condoms for anal sex in periods when you are taking PrEP?	• Always<br>• Often<br>• About half of the times I have anal sex<br>• Sometimes<br>• Never<br>• I don't know
Since I am taking PrEP, I am using condoms for anal sex…	• More often than before<br>• As often as before<br>• Less often than before<br>• I have stopped using condoms altogether<br>• I don't know
Do you indicate on your online profile(s) that you are taking PrEP?	• Yes<br>• No, but I mention it when I chat with other guys<br>• No

Wave 2

In wave 2 of the study, the following questions were added / changed:

**General information**	
What gender do you identify with?	• Male<br>• Female<br>• Trans male<br>• Trans female<br>• Inter<br>• Non-binary<br>• Other:
What gender were you assigned at birth?	• Male<br>• Female<br>• Intersexual
What is your approximate gross income per year?<br><br>*Gross income: income before deduction of taxes and social security contributions*	• Less than 30,000€<br>• 30,000 – 39,000€<br>• 40,000 – 49,000€<br>• 50,000 – 59,000€<br>• 60,000 – 69,000€<br>• 70,000€ or more<br>• I don't have any income / I am unemployed<br>• I don't know
Have you participated in this survey before?	• Yes<br>• No<br>• I don't remember

(e.g. between July and October 2018)	

What are the first three digits of the postal code of your residence in Germany?<br>If you are not living in Germany please indicate the first three digits of the postal code of the place where you are predominantly staying (e.g. hotel).<br><br>*Examples:*<br>*If your postal code is 04103, please type 041.*<br>*If your postal code is 10777, please type 107.*

**PrEP use and sexual behavior**	
Why do you take PrEP?	Select all that apply:<br>• I don't want to use condoms.<br>• I am allergic to condoms<br>• My partner doesn't want to use condoms.<br>• Sex without a condom is expected by my peers.<br>• I want to protect myself against HIV in case the condom breaks.<br>• Sometimes condoms aren't available.<br>• It's more convenient since I don't have to talk about or negotiate condom use.<br>• I don't trust my partner/s that they reliably use condoms or PrEP.<br>• I cannot get an erection or an orgasm when I use a condom.<br>• I am in an open relationship and I want to protect my partner.<br>• My partner is HIV positive.<br>• I use condoms and I want additional protection.<br>• I am less anxious during sex.<br>• Other: __________
We now have a few more questions about your sex life:<br><br>With how many different partners have you had anal and/or vaginal sex within the last 6 months?	• 0<br>• 1<br>• 2-3<br>• 4-5<br>• 6-10<br>• 11-20<br>• more than 20<br>• I don't know
Has the number of sexual partners or the number of sexual contacts increased since / when you are taking PrEP?	• Yes, I am having more partners<br>• Yes, I am having more sexual contacts<br>• Yes, I am having more partners and more contacts<br>• No<br>• I don't know
How often do you use condoms for anal / vaginal sex in periods when you are taking PrEP?	• Always<br>• Often<br>• About half of the times I have anal sex<br>• Sometimes

	• Never<br>• I don't know

Since I am taking PrEP, I am using condoms for anal / vaginal sex…	• More often than before<br>• As often as before<br>• Less often than before<br>• I have stopped using condoms altogether<br>• I don't know
Where did you obtain PrEP from?<br><br>(please indicate the last source where you obtained PrEP)	• German pharmacy – prescription for about 40-50€ (Blister prescription)<br>• German pharmacy prescription for more than 50€, but less than 100€<br>• German pharmacy – prescription for 500€ - 800€<br>• German pharmacy – my health insurance covers the costs<br>• Friends<br>• Dealer<br>• Sex Party<br>• Research Study<br>• Internet / ordered online from another country<br>• I regularly travel to another country where I get PrEP<br>• I used PEP-medication as PrEP<br>• Other: _________
Why were you not tested before starting PrEP?<br><br>Select all that apply:	• I couldn't afford the tests<br>• The tests were not offered to me<br>• I didn't have time to take the tests<br>• I didn't want to take the tests<br>• I didn't think I would benefit from these tests<br>• I didn't know I was supposed to take the tests<br>• Other reason:
Have you been tested for HIV, other sexually transmitted infections (STI), or have been checked for your kidney function **while taking PrEP?**	• Yes (all or some of the tests)<br>• No<br>• I don't know
Why are you not getting tested while taking PrEP?<br><br>Select all that apply:	• I can't afford the tests<br>• The tests were not offered to me<br>• I don't have time to take the tests<br>• I don't want to take the tests<br>• I don't think I would benefit from these tests<br>• I didn't know I was supposed to take the tests<br>• Other reason:
Where do you get tested while you are using PrEP (e.g. for HIV, STIs, or kidney function)?	• At the doctor who prescribes my PrEP<br>• At a doctor who does not prescribe my PrEP

Select all that apply:	• At a community based testing site / anonymous testing clinic<br>• Self-testing<br>• Other: ______
